# Supplementary material for: Prednisolone prescribing practices for dogs in Australia
Source: PLoS One. 2023 Feb 28;18(2):e0282440. doi: 10.1371/journal.pone.0282440 (PMC9974108; doi:10.1371/journal.pone.0282440)
Supplement: S1 Appendix — (DOCX) [file pone.0282440.s001.docx]

**Appendix 1: Body system categories for diagnoses, differentials or presenting complaints**

| **Body system** | **Includes diseases affecting the following body regions or treatment for the following clinical signs.** |
| --- | --- |
| Integument | Skin, ears, nasal planum, anal glands, pruritus |
| Ocular | Eyes |
| Respiratory | Airways and pulmonary parenchyma. Coughing, sneezing, reverse sneezing |
| Gastrointestinal | Gastrointestinal tract or oral cavity. Vomiting, diarrhoea, regurgitation |
| Neurological | Brain, spinal cord or peripheral nerves. Any neurological signs.  Spinal pain (unless primary differential is a soft tissue or skeletal injury) |
| Haematopoietic | Hemogram abnormalities, diseases of the lymph nodes, liver, or spleen. |
| Endocrine | Hypoadrenocorticism, insulinoma, hypoglycaemia |
| Musculoskeletal | Muscle, bones and joints, lameness |
| Cardiovascular | Heart worm |
| Unknown | Unclear body system affected or indication for treatment based on the medical record |
