# Supplementary material for: Prednisolone prescribing practices for dogs in Australia
Source: PLoS One. 2023 Feb 28;18(2):e0282440. doi: 10.1371/journal.pone.0282440 (PMC9974108; doi:10.1371/journal.pone.0282440)
Supplement: S2 Appendix — (DOCX) [file pone.0282440.s002.docx]

**Appendix 2: Prednisolone indicated dose categories for diagnoses, differentials or presenting complaints**

| **Indicated dose** | **Diagnosis, differential or presenting complaint** |
| --- | --- |
| Unknown | Rationale for use unclear |
| Physiologic | Hypoadrenocorticism[1]  Hypoglycaemia[1] |
| Anti-inflammatory | Inflammatory skin, ear, or ocular disease[2]  Neurological signs without differential being provided, except for tremors (see immunosuppressive).  Respiratory symptoms, such as cough or sneeze, without specific differential  Inflammatory bowel disease  Gingivitis  Urticaria[3]  Arthritis  Trauma or other soft tissue injury  Heart worm  Intervertebral disk disease[4]  Inflammatory airway disease (rhinitis, pharyngitis, laryngitis, tracheitis or bronchitis)  Tracheobronchomalacia  Syringomyelia  Neoplasia (excluding round cell neoplasia or mast cell tumours), including multiple myeloma[5]. |
| Immunosuppressive | Clinician states suspected primary immune mediated disease (undefined)  Immune mediated haemolytic anaemia[6]  Immune mediated thrombocytopenia[7]  Immune mediated polyarthritis[7]  Juvenile cellulitis[8]  Meningitis (Steroid-responsive meningitis, meningitis of unknown aetiology, granulomatous or necrotising meningitis)[9]  White shaker/Steroid responsive cerebellitis[7]  Tremors. Given common differentials are meningitis of unknown aetiology and steroid responsive cerebellitis[7]  Perianal furunculosis[10]  Lymphoma and leukaemia[5]  Mast cell tumour[11]  Lupus conditions (discoid lupus, lupus onchodystrophy)[8]  Pemphigus[12]  Vitiligo[8]  Uveodermatologic syndrome[8] |

**References**

1. Feldman EC, Nelson RW, Reusch C, Scott-Moncrieff JCR. Canine and feline endocrinology. Fourth edition. Elsevier Saunders; 2015.

2. Olivry T, Mueller RS, The International Task Force on Canine Atopic Dermatitis. Evidence-based veterinary dermatology: a systematic review of the pharmacotherapy of canine atopic dermatitis. Vet Dermatol. 2003;14: 121–146. doi:10.1046/j.1365-3164.2003.00335.x

3. Foster AP, Noli C, Rosenkrantz W. Veterinary allergy. Wiley Blackwell; 2014.

4. Sarah A. Moore, Andrea Tipold, Natasha J. Olby, Veronica Stein, Nicolas Granger, Canine Spinal Cord Injury Consortium (CANSORT SCI), et al. Current Approaches to the Management of Acute Thoracolumbar Disc Extrusion in Dogs. Front Vet Sci. 2020;7. doi:10.3389/fvets.2020.00610

5. Vail DM, Thamm DH, Liptak JM. Withrow & MacEwen’s small animal clinical oncology. Sixth edition. Elsevier; 2020.

6. Swann JW, Garden OA, Fellman CL, Glanemann B, Goggs R, LeVine DN, et al. ACVIM consensus statement on the treatment of immune-mediated hemolytic anemia in dogs. J Vet Intern Med. 2019;33: 1141–1172. doi:10.1111/jvim.15463

7. Ettinger SJ, Feldman EC, Côté E. Textbook of veterinary internal medicine : diseases of the dog and the cat. Eighth edition. Elsevier; 2017.

8. Hnilica KA, Medleau L. Small animal dermatology : a color atlas and therapeutic guide. 3rd ed. Elsevier/Saunders; 2011.

9. Cornelis I, Van Ham L, Gielen I, De Decker S, Bhatti S f. m. Clinical presentation, diagnostic findings, prognostic factors, treatment and outcome in dogs with meningoencephalomyelitis of unknown origin: A review. Vet J. 2019;244: 37–44. doi:10.1016/j.tvjl.2018.12.007

10. Cain CL. Canine Perianal Fistulas: Clinical Presentation, Pathogenesis, and Management. Vet Clin North Am Small Anim Pract. 2019;49: 53–65. doi:10.1016/j.cvsm.2018.08.006

11. Stiborova K, Treggiari E, Amores-Fuster I, del Busto I, Killick D, Maddox T, et al. Haematologic toxicity in dogs with mast cell tumours treated with vinblastine/prednisolone chemotherapy with/without radiotherapy. J Small Anim Pract. 2019;60: 534. doi:10.1111/jsap.13047

12. Tham HL, Linder KE, Olivry T. Deep pemphigus (pemphigus vulgaris, pemphigus vegetans and paraneoplastic pemphigus) in dogs, cats and horses: a comprehensive review. BMC Vet Res. 2020;16. doi:10.1186/s12917-020-02677-w
