# Supplementary material for: Prednisolone prescribing practices for dogs in Australia
Source: PLoS One. 2023 Feb 28;18(2):e0282440. doi: 10.1371/journal.pone.0282440 (PMC9974108; doi:10.1371/journal.pone.0282440)
Supplement: S3 Appendix — (DOCX) [file pone.0282440.s003.docx]

**Appendix 3: Prednisolone products prescribed to dogs in Australian veterinary practices**

| Product name | Concentration | Formulation |
| --- | --- | --- |
| Delta Cortef | 5 mg | Tablet |
| Macrolone | 20 mg | Tablet |
| Microlone | 5 mg | Tablet |
| Niralone | 5 mg | Tablet |
| Panafcortelone | 1 mg | Tablet |
| Pred-X | 5 mg and 20 mg | Tablet |
| Predmix | 5 mg/mL | Liquid (oral) |
| Prednil | 5 mg | Tablet |
| Prednisolone | 1 mg | Tablet |
| Predsolone | 1 mg | Tablet |
| Redipred | 5 mg/mL | Liquid (oral) |
| Solone | 25 mg | Tablet |
| Solu Delta Cortef | 10 mg/mL | Liquid (injectable) |
