# Supplementary material for: Prednisolone prescribing practices for dogs in Australia
Source: PLoS One. 2023 Feb 28;18(2):e0282440. doi: 10.1371/journal.pone.0282440 (PMC9974108; doi:10.1371/journal.pone.0282440)
Supplement: S2 File — (PDF) [file pone.0282440.s005.pdf]

## Generalized Linear Models

### Notes

| | | |
|---|---|---|
| Output Created | | 27-JUL-2022 10:38:37 |
| Comments | | |
| Input | Data | /Users/bonniepurcell/Desktop/SPSS /SPSS Whole data set correct sex.sav |
| | Active Dataset | DataSet1 |
| | Filter | <none> |
| | Weight | <none> |
| | Split File | <none> |
| | N of Rows in Working Data File | 2000 |
| Missing Value Handling | Definition of Missing | User-defined missing values for factor, subject and within-subject variables are treated as missing. |
| | Cases Used | Statistics are based on cases with valid data for all variables in the model. |
| Weight Handling | | not applicable |
| Syntax | | GENLIN mgkg BY bodysystemrecoded Indicateddose Sex (ORDER=ASCENDING) WITH Weight® Age_yearscalc<br>/MODEL bodysystemrecoded Indicateddose Sex Weight® Age_yearscalc INTERCEPT=YES<br>DISTRIBUTION=NORMAL LINK=IDENTITY<br>/CRITERIA SCALE=MLE COVB=MODEL PCONVERGE=1E-006 (ABSOLUTE) SINGULAR=1E-012 ANALYSISTYPE=3(LR)<br>CILEVEL=95 CITYPE=WALD LIKELIHOOD=FULL<br>/MISSING CLASSMISSING=EXCLUDE<br>/PRINT CPS DESCRIPTIVES MODELINFO FIT SUMMARY SOLUTION. |
| Resources | Processor Time | 00:00:00.62 |
| | Elapsed Time | 00:00:00.00 |

## Model Information

| | |
|---|---|
| Dependent Variable | mg/kg |
| Probability Distribution | Normal |
| Link Function | Identity |

## Case Processing Summary

| | N | Percent |
|---|---|---|
| Included | 1886 | 94.3% |
| Excluded | 114 | 5.7% |
| Total | 2000 | 100.0% |

## Categorical Variable Information

| | | | N | Percent |
|---|---|---|---|---|
| Factor | bodysystemrecoded | 2.00 | 24 | 1.3% |
| | | 3.00 | 80 | 4.2% |
| | | 4.00 | 22 | 1.2% |
| | | 5.00 | 61 | 3.2% |
| | | 6.00 | 33 | 1.7% |
| | | 7.00 | 10 | 0.5% |
| | | 8.00 | 7 | 0.4% |
| | | 10.00 | 6 | 0.3% |
| | | 11.00 | 1643 | 87.1% |
| | | Total | 1886 | 100.0% |
| | Indicated dose | 1 | 10 | 0.5% |
| | | 2 | 1796 | 95.2% |
| | | 3 | 80 | 4.2% |
| | | Total | 1886 | 100.0% |
| | Sex | 0 | 114 | 6.0% |
| | | 1 | 736 | 39.0% |
| | | 2 | 203 | 10.8% |
| | | 3 | 833 | 44.2% |
| | | Total | 1886 | 100.0% |

## Continuous Variable Information

| | | N | Minimum | Maximum | Mean |
|---|---|---|---|---|---|
| Dependent Variable | mg/kg | 1886 | .032722513 | 5.00000000 | .915651300 |
| Covariate | Weight® | 1886 | 1.56 | 90.00 | 19.3139 |
| | Age_yearscalc | 1886 | .17 | 18.33 | 6.2843 |

## Continuous Variable Information

| | | Std. Deviation |
|---|---|---|
| Dependent Variable | mg/kg | .516284623 |
| Covariate | Weight® | 12.57011 |
| | Age_yearscalc | 4.12004 |

## Goodness of Fit[a]

| | Value | df | Value/df |
|---|---|---|---|
| Deviance | 441.413 | 1871 | .236 |
| Scaled Deviance | 1886.000 | 1871 | |
| Pearson Chi-Square | 441.413 | 1871 | .236 |
| Scaled Pearson Chi-Square | 1886.000 | 1871 | |
| Log Likelihood[b] | -1306.663 | | |
| Akaike's Information Criterion (AIC) | 2645.326 | | |
| Finite Sample Corrected AIC (AICC) | 2645.617 | | |
| Bayesian Information Criterion (BIC) | 2734.002 | | |
| Consistent AIC (CAIC) | 2750.002 | | |

Dependent Variable: mg/kg
Model: (Intercept), bodysystemrecoded, Indicated dose, Sex, Weight®, Age_yearscalc[a]

a. Information criteria are in smaller-is-better form.

b. The full log likelihood function is displayed and used in computing information criteria.

## Omnibus Test[a]

| Likelihood Ratio Chi-Square | df | Sig. |
|---|---|---|
| 244.252 | 14 | .000 |

Dependent Variable: mg/kg
Model: (Intercept), bodysystemrecoded, Indicated dose, Sex, Weight®, Age_yearscalc[a]

a. Compares the fitted model against the intercept-only...

## Tests of Model Effects

| | Type III | | |
|---|---|---|---|
| Source | Likelihood Ratio Chi-Square | df | Sig. |
| (Intercept) | 394.064 | 1 | .000 |
| bodysystemrecoded | 40.236 | 7 | <.001 |
| Indicated dose | 37.182 | 1 | <.001 |
| Sex | 9.449 | 3 | .024 |
| Weight® | 30.738 | 1 | <.001 |
| Age_yearscalc | 8.751 | 1 | .003 |

Dependent Variable: mg/kg
Model: (Intercept), bodysystemrecoded, Indicated dose, Sex, Weight®, Age_yearscalc

**Parameter Estimates**

| Parameter | B | Std. Error | 95% Wald Confidence Interval | | Hypothesis Test |
|---|---|---|---|---|---|
| | | | Lower | Upper | Wald Chi-Square |
| (Intercept) | 1.468 | .0804 | 1.311 | 1.626 | 333.832 |
| [bodysystemrecoded=2.00] | .111 | .0996 | -.085 | .306 | 1.232 |
| [bodysystemrecoded=3.00] | .093 | .0573 | -.020 | .205 | 2.612 |
| [bodysystemrecoded=4.00] | .270 | .1047 | .064 | .475 | 6.629 |
| [bodysystemrecoded=5.00] | .293 | .0670 | .161 | .424 | 19.065 |
| [bodysystemrecoded=6.00] | .489 | .1116 | .270 | .707 | 19.183 |
| [bodysystemrecoded=7.00] | -1.019 | .1708 | -1.354 | -.684 | 35.597 |
| [bodysystemrecoded=8.00] | .036 | .1880 | -.333 | .404 | .036 |
| [bodysystemrecoded=10.00] | .188 | .1981 | -.201 | .576 | .898 |
| [bodysystemrecoded=11.00] | 0[a] | . | . | . | . |
| [Indicated dose=1] | 0[a] | . | . | . | . |
| [Indicated dose=2] | -.461 | .0752 | -.608 | -.313 | 37.551 |
| [Indicated dose=3] | 0[a] | . | . | . | . |
| [Sex=0] | .145 | .0487 | .050 | .241 | 8.917 |
| [Sex=1] | .011 | .0246 | -.037 | .059 | .209 |
| [Sex=2] | .042 | .0384 | -.034 | .117 | 1.175 |
| [Sex=3] | 0[a] | . | . | . | . |
| Weight® | -.005 | .0009 | -.007 | -.003 | 30.990 |
| Age_yearscalc | -.009 | .0029 | -.014 | -.003 | 8.771 |
| (Scale) | .234[b] | .0076 | .220 | .249 | |

**Parameter Estimates**

| Parameter | Hypothesis Test df | Hypothesis Test Sig. |
|---|---|---|
| (Intercept) | 1 | .000 |
| [bodysystemrecoded=2.00] | 1 | .267 |
| [bodysystemrecoded=3.00] | 1 | .106 |
| [bodysystemrecoded=4.00] | 1 | .010 |
| [bodysystemrecoded=5.00] | 1 | <.001 |
| [bodysystemrecoded=6.00] | 1 | <.001 |
| [bodysystemrecoded=7.00] | 1 | <.001 |
| [bodysystemrecoded=8.00] | 1 | .849 |
| [bodysystemrecoded=10.00] | 1 | .343 |
| [bodysystemrecoded=11.00] | . | . |
| [Indicated dose=1] | . | . |
| [Indicated dose=2] | 1 | <.001 |
| [Indicated dose=3] | . | . |
| [Sex=0] | 1 | .003 |
| [Sex=1] | 1 | .647 |
| [Sex=2] | 1 | .278 |
| [Sex=3] | . | . |
| Weight® | 1 | <.001 |
| Age_yearscalc | 1 | .003 |
| (Scale) | | |

Dependent Variable: mg/kg
Model: (Intercept), bodysystemrecoded, Indicated dose, Sex, Weight®, Age_yearscalc

a. Set to zero because this parameter is redundant.

b. Maximum likelihood estimate.

## Regression

## Notes

| | | |
|---|---|---|
| Output Created | | 27-JUL-2022 11:03:45 |
| Comments | | |
| Input | Data | /Users/bonniepurcell/Desktop/SPSS /SPSS Whole data set correct sex.sav |
| | Active Dataset | DataSet1 |
| | Filter | <none> |
| | Weight | <none> |
| | Split File | <none> |
| | N of Rows in Working Data File | 2000 |
| Missing Value Handling | Definition of Missing | User-defined missing values are treated as missing. |
| | Cases Used | Statistics are based on cases with no missing values for any variable used. |
| Syntax | | REGRESSION /MISSING LISTWISE /STATISTICS COEFF OUTS CI(95) R ANOVA CHANGE /CRITERIA=PIN(.05) POUT(.10) /NOORIGIN /DEPENDENT mgkg /METHOD=ENTER Weight® Age_yearscalc Sex Indicateddose bodysystemrecoded. |
| Resources | Processor Time | 00:00:00.05 |
| | Elapsed Time | 00:00:01.00 |
| | Memory Required | 7216 bytes |
| | Additional Memory Required for Residual Plots | 0 bytes |

## Variables Entered/Removed[a]

| Model | Variables Entered | Variables Removed | Method |
|---|---|---|---|
| 1 | bodysystemrecoded, Sex, Weight®, Indicated dose, Age_yearscalc[b] | . | Enter |

a. Dependent Variable: mg/kg

b. All requested variables entered.

## Model Summary

| Model | R | R Square | Adjusted R Square | Std. Error of the Estimate | Change Statistics | |
|---|---|---|---|---|---|---|
| | | | | | R Square Change | F Change |
| 1 | .330[a] | .109 | .107 | .487936741 | .109 | 46.078 |

## Model Summary

| Model | Change Statistics | | |
|---|---|---|---|
| | df1 | df2 | Sig. F Change |
| 1 | 5 | 1880 | <.001 |

a. Predictors: (Constant), bodysystemrecoded, Sex, Weight®, Indicated dose, Age_yearscalc

## ANOVA[a]

| Model | | Sum of Squares | df | Mean Square | F | Sig. |
|---|---|---|---|---|---|---|
| 1 | Regression | 54.852 | 5 | 10.970 | 46.078 | <.001[b] |
| | Residual | 447.595 | 1880 | .238 | | |
| | Total | 502.446 | 1885 | | | |

a. Dependent Variable: mg/kg

b. Predictors: (Constant), bodysystemrecoded, Sex, Weight®, Indicated dose, Age_yearscalc

## Coefficients[a]

| Model | | Unstandardized Coefficients | | Standardized Coefficients | t | Sig. |
|---|---|---|---|---|---|---|
| | | B | Std. Error | Beta | | |
| 1 | (Constant) | .032 | .134 | | .241 | .809 |
| | Weight® | -.005 | .001 | -.120 | -5.348 | <.001 |
| | Age_yearscalc | -.009 | .003 | -.074 | -3.195 | .001 |
| | Sex | -.019 | .011 | -.039 | -1.776 | .076 |
| | Indicated dose | .644 | .053 | .268 | 12.107 | <.001 |
| | bodysystemrecoded | -.023 | .005 | -.105 | -4.515 | <.001 |

## Coefficients[a]

| Model | | 95.0% Confidence Interval for B | |
|---|---|---|---|
| | | Lower Bound | Upper Bound |
| 1 | (Constant) | -.231 | .296 |
| | Weight® | -.007 | -.003 |
| | Age_yearscalc | -.015 | -.004 |
| | Sex | -.041 | .002 |
| | Indicated dose | .539 | .748 |
| | bodysystemrecoded | -.034 | -.013 |

a. Dependent Variable: mg/kg

## Generalized Linear Models

### Notes

| | | |
|---|---|---|
| Output Created | | 27-JUL-2022 11:09:50 |
| Comments | | |
| Input | Data | /Users/bonniepurcell/Desktop/SPSS /SPSS Whole data set correct sex.sav |
| | Active Dataset | DataSet1 |
| | Filter | <none> |
| | Weight | <none> |
| | Split File | <none> |
| | N of Rows in Working Data File | 2000 |
| Missing Value Handling | Definition of Missing | User-defined missing values for factor, subject and within-subject variables are treated as missing. |
| | Cases Used | Statistics are based on cases with valid data for all variables in the model. |
| Weight Handling | | not applicable |
| Syntax | | GENLIN mgm2 BY bodysystemrecoded Indicateddose Sex (ORDER=ASCENDING) WITH Weight® Age_yearscalc<br>/MODEL bodysystemrecoded Indicateddose Sex Weight® Age_yearscalc INTERCEPT=YES<br>DISTRIBUTION=NORMAL LINK=IDENTITY<br>/CRITERIA SCALE=MLE COVB=MODEL PCONVERGE=1E-006 (ABSOLUTE) SINGULAR=1E-012 ANALYSISTYPE=3(LR)<br>CILEVEL=95 CITYPE=WALD LIKELIHOOD=FULL<br>/MISSING CLASSMISSING=EXCLUDE<br>/PRINT CPS DESCRIPTIVES MODELINFO FIT SUMMARY SOLUTION. |
| Resources | Processor Time | 00:00:00.63 |
| | Elapsed Time | 00:00:01.00 |

### Model Information

| | |
|---|---|
| Dependent Variable | mg/m2 |
| Probability Distribution | Normal |
| Link Function | Identity |

### Case Processing Summary

| | N | Percent |
|---|---|---|
| Included | 1886 | 94.3% |
| Excluded | 114 | 5.7% |
| Total | 2000 | 100.0% |

## Generalized Linear Models

### Notes

| | | |
|---|---|---|
| Output Created | | 27-JUL-2022 11:56:51 |
| Comments | | |
| Input | Data | /Users/bonniepurcell/Desktop/SPSS /SPSS Whole data set correct sex.sav |
| | Active Dataset | DataSet1 |
| | Filter | <none> |
| | Weight | <none> |
| | Split File | <none> |
| | N of Rows in Working Data File | 2000 |
| Missing Value Handling | Definition of Missing | User-defined missing values for factor, subject and within-subject variables are treated as missing. |
| | Cases Used | Statistics are based on cases with valid data for all variables in the model. |
| Weight Handling | | not applicable |

## Notes

| | | |
|---|---|---|
| Syntax | | GENLIN mgm2 BY bodysystemrecoded Indicateddose (ORDER=ASCENDING) WITH Weight® Age_yearscalc /MODEL bodysystemrecoded Indicateddose Weight® Age_yearscalc INTERCEPT=YES DISTRIBUTION=NORMAL LINK=IDENTITY /CRITERIA SCALE=MLE COVB=MODEL PCONVERGE=1E-006 (ABSOLUTE) SINGULAR=1E-012 ANALYSISTYPE=3(LR) CILEVEL=95 CITYPE=WALD LIKELIHOOD=FULL /MISSING CLASSMISSING=EXCLUDE /PRINT CPS DESCRIPTIVES MODELINFO FIT SUMMARY SOLUTION. |
| Resources | Processor Time | 00:00:00.42 |
| | Elapsed Time | 00:00:00.00 |

## Model Information

| | |
|---|---|
| Dependent Variable | mg/m2 |
| Probability Distribution | Normal |
| Link Function | Identity |

## Case Processing Summary

| | N | Percent |
|---|---|---|
| Included | 1886 | 94.3% |
| Excluded | 114 | 5.7% |
| Total | 2000 | 100.0% |

## Categorical Variable Information

| | | | N | Percent |
|---|---|---|---|---|
| Factor | bodysystemrecoded | 2.00 | 24 | 1.3% |
| | | 3.00 | 80 | 4.2% |
| | | 4.00 | 22 | 1.2% |
| | | 5.00 | 61 | 3.2% |
| | | 6.00 | 33 | 1.7% |
| | | 7.00 | 10 | 0.5% |
| | | 8.00 | 7 | 0.4% |
| | | 10.00 | 6 | 0.3% |
| | | 11.00 | 1643 | 87.1% |
| | | Total | 1886 | 100.0% |
| | Indicated dose | 1 | 10 | 0.5% |
| | | 2 | 1796 | 95.2% |
| | | 3 | 80 | 4.2% |
| | | Total | 1886 | 100.0% |

## Continuous Variable Information

| | | N | Minimum | Maximum | Mean |
|---|---|---|---|---|---|
| Dependent Variable | mg/m2 | 1886 | 1.09113813 | 106.655183 | 22.7675797 |
| Covariate | Weight® | 1886 | 1.56 | 90.00 | 19.3139 |
| | Age_yearscalc | 1886 | .17 | 18.33 | 6.2843 |

## Continuous Variable Information

| | | Std. Deviation |
|---|---|---|
| Dependent Variable | mg/m2 | 13.0648639 |
| Covariate | Weight® | 12.57011 |
| | Age_yearscalc | 4.12004 |

## Goodness of Fit[a]

| | Value | df | Value/df |
|---|---|---|---|
| Deviance | 270631.591 | 1874 | 144.414 |
| Scaled Deviance | 1886.000 | 1874 | |
| Pearson Chi-Square | 270631.591 | 1874 | 144.414 |
| Scaled Pearson Chi-Square | 1886.000 | 1874 | |
| Log Likelihood[b] | -7359.339 | | |
| Akaike's Information Criterion (AIC) | 14744.678 | | |
| Finite Sample Corrected AIC (AICC) | 14744.873 | | |
| Bayesian Information Criterion (BIC) | 14816.727 | | |
| Consistent AIC (CAIC) | 14829.727 | | |

Dependent Variable: mg/m2
Model: (Intercept), bodysystemrecoded, Indicated dose, Weight®, Age_yearscalc[a]

a. Information criteria are in smaller-is-better form.

b. The full log likelihood function is displayed and used in computing information criteria.

## Omnibus Test[a]

| Likelihood Ratio Chi-Square | df | Sig. |
|---|---|---|
| 326.320 | 11 | .000 |

Dependent Variable: mg/m2
Model: (Intercept), bodysystemrecoded, Indicated dose, Weight®, Age_yearscalc[a]

a. Compares the fitted model against the intercept-only...

## Tests of Model Effects

| | Type III | | |
|---|---|---|---|
| Source | Likelihood Ratio Chi-Square | df | Sig. |
| (Intercept) | 393.523 | 1 | .000 |
| bodysystemrecoded | 24.993 | 7 | <.001 |
| Indicated dose | 37.137 | 1 | <.001 |
| Weight® | 138.359 | 1 | .000 |
| Age_yearscalc | 11.390 | 1 | <.001 |

Dependent Variable: mg/m2
Model: (Intercept), bodysystemrecoded, Indicated dose, Weight®, Age_yearscalc

## Parameter Estimates

| Parameter | B | Std. Error | 95% Wald Confidence Interval | | Hypothesis Test |
|---|---|---|---|---|---|
| | | | Lower | Upper | Wald Chi-Square |
| (Intercept) | 29.489 | 1.9592 | 25.649 | 33.329 | 226.556 |
| [bodysystemrecoded=2.00] | 2.466 | 2.4648 | -2.365 | 7.297 | 1.001 |
| [bodysystemrecoded=3.00] | 1.657 | 1.4181 | -1.122 | 4.437 | 1.366 |
| [bodysystemrecoded=4.00] | 4.776 | 2.5896 | -.299 | 9.852 | 3.402 |
| [bodysystemrecoded=5.00] | 4.556 | 1.6540 | 1.315 | 7.798 | 7.589 |
| [bodysystemrecoded=6.00] | 11.231 | 2.7617 | 5.818 | 16.644 | 16.538 |
| [bodysystemrecoded=7.00] | -24.155 | 4.2257 | -32.438 | -15.873 | 32.676 |
| [bodysystemrecoded=8.00] | 4.453 | 4.6550 | -4.671 | 13.576 | .915 |
| [bodysystemrecoded=10.00] | 3.178 | 4.9032 | -6.432 | 12.788 | .420 |
| [bodysystemrecoded=11.00] | 0[a] | . | . | . | . |
| [Indicated dose=1] | 0[a] | . | . | . | . |
| [Indicated dose=2] | -11.390 | 1.8598 | -15.035 | -7.744 | 37.505 |
| [Indicated dose=3] | 0[a] | . | . | . | . |
| Weight® | .271 | .0226 | .227 | .316 | 143.561 |
| Age_yearscalc | -.241 | .0712 | -.380 | -.101 | 11.424 |
| (Scale) | 143.495[b] | 4.6728 | 134.623 | 152.952 | |

**Parameter Estimates**

| Parameter | Hypothesis Test df | Sig. |
|---|---|---|
| (Intercept) | 1 | .000 |
| [bodysystemrecoded=2.00] | 1 | .317 |
| [bodysystemrecoded=3.00] | 1 | .243 |
| [bodysystemrecoded=4.00] | 1 | .065 |
| [bodysystemrecoded=5.00] | 1 | .006 |
| [bodysystemrecoded=6.00] | 1 | <.001 |
| [bodysystemrecoded=7.00] | 1 | <.001 |
| [bodysystemrecoded=8.00] | 1 | .339 |
| [bodysystemrecoded=10.00] | 1 | .517 |
| [bodysystemrecoded=11.00] | . | . |
| [Indicated dose=1] | . | . |
| [Indicated dose=2] | 1 | <.001 |
| [Indicated dose=3] | . | . |
| Weight® | 1 | .000 |
| Age_yearscalc | 1 | <.001 |
| (Scale) | | |

Dependent Variable: mg/m2
Model: (Intercept), bodysystemrecoded, Indicated dose, Weight®, Age_yearscalc

a. Set to zero because this parameter is redundant.

b. Maximum likelihood estimate.
