# Supplementary material for: Prednisolone prescribing practices for dogs in Australia
Source: PLoS One. 2023 Feb 28;18(2):e0282440. doi: 10.1371/journal.pone.0282440 (PMC9974108; doi:10.1371/journal.pone.0282440)
Supplement: S3 File — (PDF) [file pone.0282440.s006.pdf]

Generalized Linear Models

Notes

Output Created		13-JUL-2022 17:13:08
Comments		
Input	Data	/Users/bonniepurcell/Desktop/SPSS /SPSS Whole data set correct sex.sav
	Active Dataset	DataSet1
	Filter	<none>
	Weight	<none>
	Split File	<none>
	N of Rows in Working Data File	2000
Missing Value Handling	Definition of Missing	User-defined missing values for factor, subject and within-subject variables are treated as missing.
	Cases Used	Statistics are based on cases with valid data for all variables in the model.
Weight Handling		not applicable
Syntax		GENLIN mgkg BY bodysystemrecoded Indicateddose Sex (ORDER=ASCENDING) WITH Agemonths Weight® /MODEL bodysystemrecoded Indicateddose Sex Agemonths Weight® INTERCEPT=YES DISTRIBUTION=NORMAL LINK=IDENTITY /CRITERIA SCALE=MLE COVB=MODEL PCONVERGE=1E-006 (ABSOLUTE) SINGULAR=1E-012 ANALYSISTYPE=3(LR) CILEVEL=95 CITYPE=WALD LIKELIHOOD=FULL /MISSING CLASSMISSING=EXCLUDE /PRINT CPS DESCRIPTIVES MODELINFO FIT SUMMARY SOLUTION.
Resources	Processor Time	00:00:00.65
	Elapsed Time	00:00:01.00

```
[DataSet1] /Users/bonniepurcell/Desktop/SPSS /SPSS Whole data set correct sex.sav
```

Model Information

Dependent Variable	mg/kg
Probability Distribution	Normal
Link Function	Identity

Case Processing Summary

	N	Percent
Included	1886	94.3%
Excluded	114	5.7%
Total	2000	100.0%

Categorical Variable Information

			N	Percent
Factor	bodysystemrecoded	2.00	24	1.3%
		3.00	80	4.2%
		4.00	22	1.2%
		5.00	61	3.2%
		6.00	33	1.7%
		7.00	10	0.5%
		8.00	7	0.4%
		10.00	6	0.3%
		11.00	1643	87.1%
		Total	1886	100.0%
	Indicated dose	1	10	0.5%
		2	1796	95.2%
		3	80	4.2%
		Total	1886	100.0%
	Sex	0	114	6.0%
		1	736	39.0%
		2	203	10.8%
		3	833	44.2%
		Total	1886	100.0%

Continuous Variable Information

		N	Minimum	Maximum	Mean
Dependent Variable	mg/kg	1886	.032722513	5.00000000	.915651300
Covariate	Age (months)	1886	2	220	75.41
	Weight®	1886	1.56	90.00	19.3139

Continuous Variable Information

		Std. Deviation
Dependent Variable	mg/kg	.516284623
Covariate	Age (months)	49.441
	Weight®	12.57011

Goodness of Fit[a]

	Value	df	Value/df
Deviance	441.413	1871	.236
Scaled Deviance	1886.000	1871	
Pearson Chi-Square	441.413	1871	.236
Scaled Pearson Chi-Square	1886.000	1871	
Log Likelihood[b]	-1306.663		
Akaike's Information Criterion (AIC)	2645.326		
Finite Sample Corrected AIC (AICC)	2645.617		
Bayesian Information Criterion (BIC)	2734.002		
Consistent AIC (CAIC)	2750.002		

Dependent Variable: mg/kg
Model: (Intercept), bodysystemrecoded, Indicated dose, Sex, Age (months), Weight®[a]

a. Information criteria are in smaller-is-better form.

b. The full log likelihood function is displayed and used in computing information criteria.

Omnibus Test[a]

Likelihood Ratio Chi-Square	df	Sig.
244.252	14	.000

Dependent Variable: mg/kg
Model: (Intercept), bodysystemrecoded, Indicated dose, Sex, Age (months), Weight®[a]

a. Compares the fitted model against the intercept-only...

Tests of Model Effects

	Type III		
Source	Likelihood Ratio Chi-Square	df	Sig.
(Intercept)	394.064	1	.000
bodysystemrecoded	40.236	7	<.001
Indicated dose	37.182	1	<.001
Sex	9.449	3	.024
Age (months)	8.751	1	.003
Weight®	30.738	1	<.001

Dependent Variable: mg/kg
Model: (Intercept), bodysystemrecoded, Indicated dose, Sex, Age (months), Weight®

Parameter Estimates

Parameter	B	Std. Error	95% Wald Confidence Interval		Hypothesis Test
			Lower	Upper	Wald Chi-Square
(Intercept)	1.468	.0804	1.311	1.626	333.832
[bodysystemrecoded=2.00]	.111	.0996	-.085	.306	1.232
[bodysystemrecoded=3.00]	.093	.0573	-.020	.205	2.612
[bodysystemrecoded=4.00]	.270	.1047	.064	.475	6.629
[bodysystemrecoded=5.00]	.293	.0670	.161	.424	19.065
[bodysystemrecoded=6.00]	.489	.1116	.270	.707	19.183
[bodysystemrecoded=7.00]	-1.019	.1708	-1.354	-.684	35.597
[bodysystemrecoded=8.00]	.036	.1880	-.333	.404	.036
[bodysystemrecoded=10.00]	.188	.1981	-.201	.576	.898
[bodysystemrecoded=11.00]	0[a]	.	.	.	.
[Indicated dose=1]	0[a]	.	.	.	.
[Indicated dose=2]	-.461	.0752	-.608	-.313	37.551
[Indicated dose=3]	0[a]	.	.	.	.
[Sex=0]	.145	.0487	.050	.241	8.917
[Sex=1]	.011	.0246	-.037	.059	.209
[Sex=2]	.042	.0384	-.034	.117	1.175
[Sex=3]	0[a]	.	.	.	.
Age (months)	-.001	.0002	-.001	.000	8.771
Weight®	-.005	.0009	-.007	-.003	30.990
(Scale)	.234[b]	.0076	.220	.249	

Parameter Estimates

Parameter	Hypothesis Test	
	df	Sig.
(Intercept)	1	.000
[bodysystemrecoded=2.00]	1	.267
[bodysystemrecoded=3.00]	1	.106
[bodysystemrecoded=4.00]	1	.010
[bodysystemrecoded=5.00]	1	<.001
[bodysystemrecoded=6.00]	1	<.001
[bodysystemrecoded=7.00]	1	<.001
[bodysystemrecoded=8.00]	1	.849
[bodysystemrecoded=10.00]	1	.343
[bodysystemrecoded=11.00]	.	.
[Indicated dose=1]	.	.
[Indicated dose=2]	1	<.001
[Indicated dose=3]	.	.
[Sex=0]	1	.003
[Sex=1]	1	.647
[Sex=2]	1	.278
[Sex=3]	.	.
Age (months)	1	.003
Weight®	1	<.001
(Scale)		

Dependent Variable: mg/kg
Model: (Intercept), bodysystemrecoded, Indicated dose, Sex, Age (months), Weight®

a. Set to zero because this parameter is redundant.

b. Maximum likelihood estimate.

Generalized Linear Models

Notes

Output Created		13-JUL-2022 17:18:54
Comments		
Input	Data	/Users/bonniepurcell/Desktop/SPSS /SPSS Whole data set correct sex.sav
	Active Dataset	DataSet1
	Filter	<none>
	Weight	<none>
	Split File	<none>
	N of Rows in Working Data File	2000
Missing Value Handling	Definition of Missing	User-defined missing values for factor, subject and within-subject variables are treated as missing.
	Cases Used	Statistics are based on cases with valid data for all variables in the model.
Weight Handling		not applicable
Syntax		GENLIN mgkg BY bodysystemrecoded Indicateddose Sex (ORDER=ASCENDING) WITH Agemonths Weight® /MODEL bodysystemrecoded Indicateddose Sex Agemonths Weight® INTERCEPT=YES DISTRIBUTION=NORMAL LINK=IDENTITY /CRITERIA SCALE=MLE COVB=MODEL PCONVERGE=1E-006 (ABSOLUTE) SINGULAR=1E-012 ANALYSISTYPE=3(LR) CILEVEL=95 CITYPE=WALD LIKELIHOOD=FULL /MISSING CLASSMISSING=EXCLUDE /PRINT CPS DESCRIPTIVES MODELINFO FIT SUMMARY SOLUTION /SAVE MEANPRED RESID PEARSONRESID STDPEARSONRESID.
Resources	Processor Time	00:00:00.71
	Elapsed Time	00:00:00.00
Variables Created or Modified	Predicted Value of the Mean of the Response	MeanPredicted
	Raw Residual	Residual

Notes

	Pearson Residual	PearsonResidual
	Standardized Pearson Residual	StdPearsonResidual

Model Information

Dependent Variable	mg/kg
Probability Distribution	Normal
Link Function	Identity

Case Processing Summary

	N	Percent
Included	1886	94.3%
Excluded	114	5.7%
Total	2000	100.0%

Categorical Variable Information

			N	Percent
Factor	bodysystemrecoded	2.00	24	1.3%
		3.00	80	4.2%
		4.00	22	1.2%
		5.00	61	3.2%
		6.00	33	1.7%
		7.00	10	0.5%
		8.00	7	0.4%
		10.00	6	0.3%
		11.00	1643	87.1%
		Total	1886	100.0%
	Indicated dose	1	10	0.5%
		2	1796	95.2%
		3	80	4.2%
		Total	1886	100.0%
	Sex	0	114	6.0%
		1	736	39.0%
		2	203	10.8%
		3	833	44.2%
		Total	1886	100.0%

Continuous Variable Information

		N	Minimum	Maximum	Mean
Dependent Variable	mg/kg	1886	.032722513	5.00000000	.915651300
Covariate	Age (months)	1886	2	220	75.41
	Weight®	1886	1.56	90.00	19.3139

Continuous Variable Information

		Std. Deviation
Dependent Variable	mg/kg	.516284623
Covariate	Age (months)	49.441
	Weight®	12.57011

Goodness of Fit[a]

	Value	df	Value/df
Deviance	441.413	1871	.236
Scaled Deviance	1886.000	1871	
Pearson Chi-Square	441.413	1871	.236
Scaled Pearson Chi-Square	1886.000	1871	
Log Likelihood[b]	-1306.663		
Akaike's Information Criterion (AIC)	2645.326		
Finite Sample Corrected AIC (AICC)	2645.617		
Bayesian Information Criterion (BIC)	2734.002		
Consistent AIC (CAIC)	2750.002		

Dependent Variable: mg/kg
Model: (Intercept), bodysystemrecoded, Indicated dose, Sex, Age (months), Weight®[a]

a. Information criteria are in smaller-is-better form.

b. The full log likelihood function is displayed and used in computing information criteria.

Omnibus Test[a]

Likelihood Ratio Chi-Square	df	Sig.
244.252	14	.000

Dependent Variable: mg/kg
Model: (Intercept), bodysystemrecoded, Indicated dose, Sex, Age (months), Weight®[a]

a. Compares the fitted model against the intercept-only...

Tests of Model Effects

Source	Type III		
	Likelihood Ratio Chi-Square	df	Sig.
(Intercept)	394.064	1	.000
bodysystemrecoded	40.236	7	<.001
Indicated dose	37.182	1	<.001
Sex	9.449	3	.024
Age (months)	8.751	1	.003
Weight®	30.738	1	<.001

Dependent Variable: mg/kg
Model: (Intercept), bodysystemrecoded, Indicated dose, Sex, Age (months), Weight®

Parameter Estimates

Parameter	B	Std. Error	95% Wald Confidence Interval		Hypothesis Test
			Lower	Upper	Wald Chi-Square
(Intercept)	1.468	.0804	1.311	1.626	333.832
[bodysystemrecoded=2.00]	.111	.0996	-.085	.306	1.232
[bodysystemrecoded=3.00]	.093	.0573	-.020	.205	2.612
[bodysystemrecoded=4.00]	.270	.1047	.064	.475	6.629
[bodysystemrecoded=5.00]	.293	.0670	.161	.424	19.065
[bodysystemrecoded=6.00]	.489	.1116	.270	.707	19.183
[bodysystemrecoded=7.00]	-1.019	.1708	-1.354	-.684	35.597
[bodysystemrecoded=8.00]	.036	.1880	-.333	.404	.036
[bodysystemrecoded=10.00]	.188	.1981	-.201	.576	.898
[bodysystemrecoded=11.00]	0[a]	.	.	.	.
[Indicated dose=1]	0[a]	.	.	.	.
[Indicated dose=2]	-.461	.0752	-.608	-.313	37.551
[Indicated dose=3]	0[a]	.	.	.	.
[Sex=0]	.145	.0487	.050	.241	8.917
[Sex=1]	.011	.0246	-.037	.059	.209
[Sex=2]	.042	.0384	-.034	.117	1.175
[Sex=3]	0[a]	.	.	.	.
Age (months)	-.001	.0002	-.001	.000	8.771
Weight®	-.005	.0009	-.007	-.003	30.990
(Scale)	.234[b]	.0076	.220	.249	

Parameter Estimates

Parameter	Hypothesis Test df	Sig.
(Intercept)	1	.000
[bodysystemrecoded=2.00]	1	.267
[bodysystemrecoded=3.00]	1	.106
[bodysystemrecoded=4.00]	1	.010
[bodysystemrecoded=5.00]	1	<.001
[bodysystemrecoded=6.00]	1	<.001
[bodysystemrecoded=7.00]	1	<.001
[bodysystemrecoded=8.00]	1	.849
[bodysystemrecoded=10.00]	1	.343
[bodysystemrecoded=11.00]	.	.
[Indicated dose=1]	.	.
[Indicated dose=2]	1	<.001
[Indicated dose=3]	.	.
[Sex=0]	1	.003
[Sex=1]	1	.647
[Sex=2]	1	.278
[Sex=3]	.	.
Age (months)	1	.003
Weight®	1	<.001
(Scale)		

Dependent Variable: mg/kg
Model: (Intercept), bodysystemrecoded, Indicated dose, Sex, Age (months), Weight®

a. Set to zero because this parameter is redundant.

b. Maximum likelihood estimate.

GGraph

Notes

Output Created		13-JUL-2022 17:20:38
Comments		
Input	Data	/Users/bonniepurcell/Desktop/SPSS /SPSS Whole data set correct sex.sav
	Active Dataset	DataSet1
	Filter	<none>
	Weight	<none>
	Split File	<none>
	N of Rows in Working Data File	2000
Syntax		GGRAPH<br>/GRAPHDATASET NAME="graphdataset" VARIABLES=Residual MISSING=LISTWISE REPORTMISSING=NO<br>/GRAPHSPEC SOURCE=INLINE.<br>BEGIN GPL<br>SOURCE: s=userSource(id("graphdataset"))<br>DATA: Residual=col(source(s), name("Residual"))<br>GUIDE: axis(dim(1), label("Raw Residual"))<br>GUIDE: axis(dim(2), label("Frequency"))<br>GUIDE: text.title(label("Simple Histogram of Raw Residual"))<br>ELEMENT: interval(position(summary.count(bin.rect(Residual))), shape.interior(shape.square))<br>END GPL.
Resources	Processor Time	00:00:02.92
	Elapsed Time	00:00:03.00

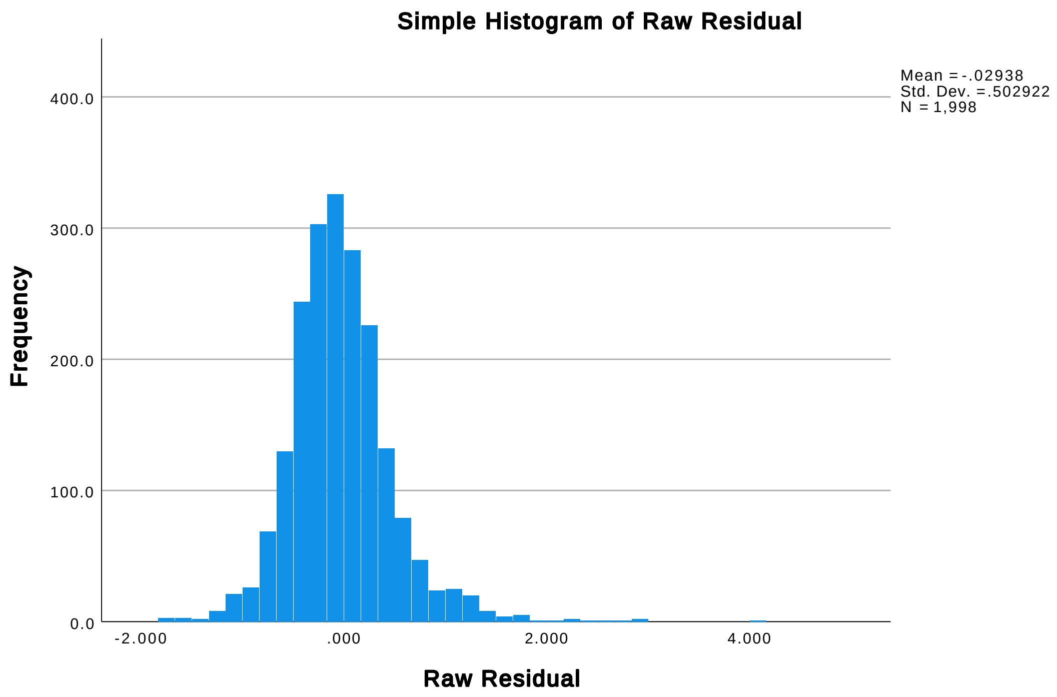

GGraph

Notes

Output Created		13-JUL-2022 17:22:54
Comments		
Input	Data	/Users/bonniepurcell/Desktop/SPSS /SPSS Whole data set correct sex.sav
	Active Dataset	DataSet1
	Filter	<none>
	Weight	<none>
	Split File	<none>
	N of Rows in Working Data File	2000

Notes

Syntax		GGRAPH<br>/GRAPHDATASET NAME="graphdataset" VARIABLES=MeanPredicted mgkg MISSING=LISTWISE REPORTMISSING=NO<br>/GRAPHSPEC SOURCE=INLINE<br>/FITLINE TOTAL=NO SUBGROUP=NO.<br>BEGIN GPL<br>SOURCE: s=userSource (id("graphdataset"))<br>DATA: MeanPredicted=col (source(s), name ("MeanPredicted"))<br>DATA: mgkg=col (source(s), name("mgkg"))<br>GUIDE: axis(dim(1), label("Predicted Value of Mean of Response"))<br>GUIDE: axis(dim(2), label("mg/kg"))<br>GUIDE: text.title(label ("Scatter Plot of mg/kg by Predicted Value of Mean of Response"))<br>ELEMENT: point(position (MeanPredicted*mgkg))<br>END GPL.
Resources	Processor Time	00:00:00.21
	Elapsed Time	00:00:00.00

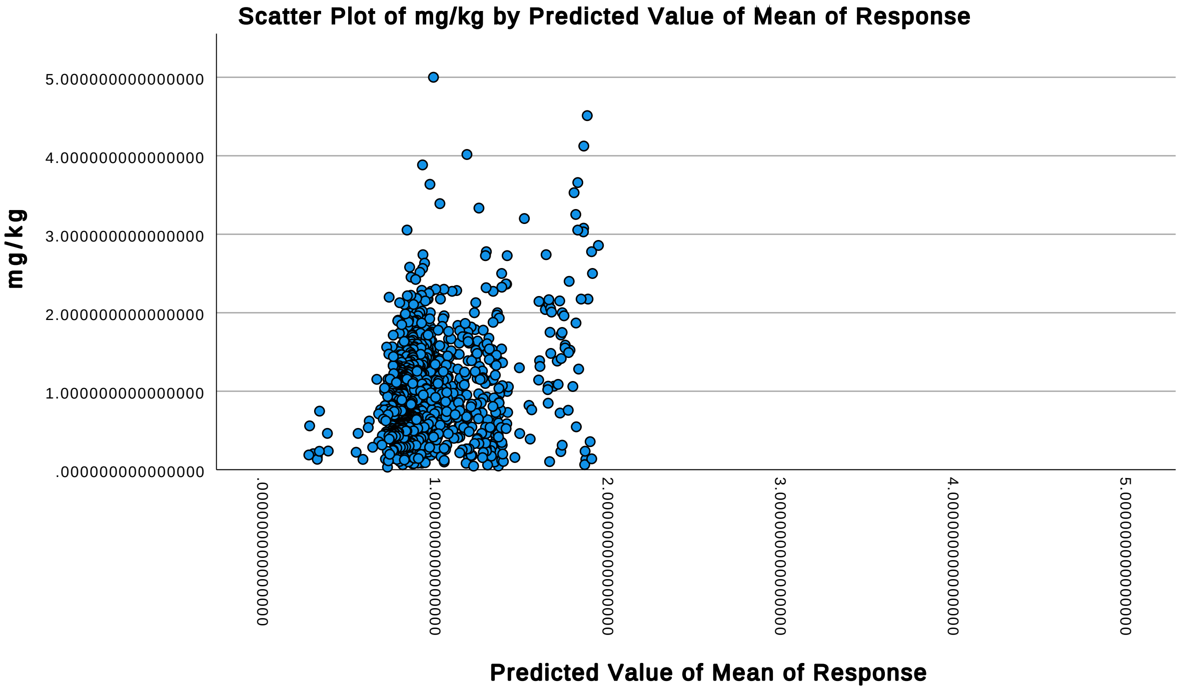

Generalized Linear Models

Notes

Output Created		13-JUL-2022 17:28:05
Comments		
Input	Data	/Users/bonniepurcell/Desktop/SPSS /SPSS Whole data set correct sex.sav
	Active Dataset	DataSet1
	Filter	<none>
	Weight	<none>
	Split File	<none>
	N of Rows in Working Data File	2000
Missing Value Handling	Definition of Missing	User-defined missing values for factor, subject and within-subject variables are treated as missing.
	Cases Used	Statistics are based on cases with valid data for all variables in the model.
Weight Handling		not applicable
Syntax		GENLIN mgkg BY bodysystemrecoded Indicateddose Sex (ORDER=ASCENDING) WITH Agemonths Weight® /MODEL bodysystemrecoded Indicateddose Sex Agemonths Weight® INTERCEPT=YES DISTRIBUTION=NORMAL LINK=IDENTITY /CRITERIA SCALE=MLE COVB=MODEL PCONVERGE=1E-006 (ABSOLUTE) SINGULAR=1E-012 ANALYSISTYPE=3(LR) CILEVEL=95 CITYPE=WALD LIKELIHOOD=FULL /MISSING CLASSMISSING=EXCLUDE /PRINT CPS DESCRIPTIVES MODELINFO FIT SUMMARY SOLUTION /SAVE MEANPRED COOK RESID PEARSONRESID STDPEARSONRESID.
Resources	Processor Time	00:00:00.71
	Elapsed Time	00:00:01.00

Notes

Variables Created or Modified	Predicted Value of the Mean of the Response	MeanPredicted_1
	Raw Residual	Residual_1
	Pearson Residual	PearsonResidual_1
	Standardized Pearson Residual	StdPearsonResidual_1
	Cook's Distance	CooksDistance

Model Information

Dependent Variable	mg/kg
Probability Distribution	Normal
Link Function	Identity

Case Processing Summary

	N	Percent
Included	1886	94.3%
Excluded	114	5.7%
Total	2000	100.0%

Categorical Variable Information

			N	Percent
Factor	bodysystemrecoded	2.00	24	1.3%
		3.00	80	4.2%
		4.00	22	1.2%
		5.00	61	3.2%
		6.00	33	1.7%
		7.00	10	0.5%
		8.00	7	0.4%
		10.00	6	0.3%
		11.00	1643	87.1%
		Total	1886	100.0%
	Indicated dose	1	10	0.5%
		2	1796	95.2%
		3	80	4.2%
		Total	1886	100.0%
	Sex	0	114	6.0%
		1	736	39.0%
		2	203	10.8%
		3	833	44.2%
		Total	1886	100.0%

Continuous Variable Information

		N	Minimum	Maximum	Mean
Dependent Variable	mg/kg	1886	.032722513	5.00000000	.915651300
Covariate	Age (months)	1886	2	220	75.41
	Weight®	1886	1.56	90.00	19.3139

Continuous Variable Information

		Std. Deviation
Dependent Variable	mg/kg	.516284623
Covariate	Age (months)	49.441
	Weight®	12.57011

Goodness of Fit[a]

	Value	df	Value/df
Deviance	441.413	1871	.236
Scaled Deviance	1886.000	1871	
Pearson Chi-Square	441.413	1871	.236
Scaled Pearson Chi-Square	1886.000	1871	
Log Likelihood[b]	-1306.663		
Akaike's Information Criterion (AIC)	2645.326		
Finite Sample Corrected AIC (AICC)	2645.617		
Bayesian Information Criterion (BIC)	2734.002		
Consistent AIC (CAIC)	2750.002		

Dependent Variable: mg/kg
Model: (Intercept), bodysystemrecoded, Indicated dose, Sex, Age (months), Weight®[a]

a. Information criteria are in smaller-is-better form.

b. The full log likelihood function is displayed and used in computing information criteria.

Omnibus Test[a]

Likelihood Ratio Chi-Square	df	Sig.
244.252	14	.000

Dependent Variable: mg/kg
Model: (Intercept), bodysystemrecoded, Indicated dose, Sex, Age (months), Weight®[a]

a. Compares the fitted model against the intercept-only...

Tests of Model Effects

Source	Type III		
	Likelihood Ratio Chi-Square	df	Sig.
(Intercept)	394.064	1	.000
bodysystemrecoded	40.236	7	<.001
Indicated dose	37.182	1	<.001
Sex	9.449	3	.024
Age (months)	8.751	1	.003
Weight®	30.738	1	<.001

Dependent Variable: mg/kg
Model: (Intercept), bodysystemrecoded, Indicated dose, Sex, Age (months), Weight®

Parameter Estimates

Parameter	B	Std. Error	95% Wald Confidence Interval		Hypothesis Test
			Lower	Upper	Wald Chi-Square
(Intercept)	1.468	.0804	1.311	1.626	333.832
[bodysystemrecoded=2.00]	.111	.0996	-.085	.306	1.232
[bodysystemrecoded=3.00]	.093	.0573	-.020	.205	2.612
[bodysystemrecoded=4.00]	.270	.1047	.064	.475	6.629
[bodysystemrecoded=5.00]	.293	.0670	.161	.424	19.065
[bodysystemrecoded=6.00]	.489	.1116	.270	.707	19.183
[bodysystemrecoded=7.00]	-1.019	.1708	-1.354	-.684	35.597
[bodysystemrecoded=8.00]	.036	.1880	-.333	.404	.036
[bodysystemrecoded=10.00]	.188	.1981	-.201	.576	.898
[bodysystemrecoded=11.00]	0[a]	.	.	.	.
[Indicated dose=1]	0[a]	.	.	.	.
[Indicated dose=2]	-.461	.0752	-.608	-.313	37.551
[Indicated dose=3]	0[a]	.	.	.	.
[Sex=0]	.145	.0487	.050	.241	8.917
[Sex=1]	.011	.0246	-.037	.059	.209
[Sex=2]	.042	.0384	-.034	.117	1.175
[Sex=3]	0[a]	.	.	.	.
Age (months)	-.001	.0002	-.001	.000	8.771
Weight®	-.005	.0009	-.007	-.003	30.990
(Scale)	.234[b]	.0076	.220	.249	

Parameter Estimates

Parameter	Hypothesis Test df	Sig.
(Intercept)	1	.000
[bodysystemrecoded=2.00]	1	.267
[bodysystemrecoded=3.00]	1	.106
[bodysystemrecoded=4.00]	1	.010
[bodysystemrecoded=5.00]	1	<.001
[bodysystemrecoded=6.00]	1	<.001
[bodysystemrecoded=7.00]	1	<.001
[bodysystemrecoded=8.00]	1	.849
[bodysystemrecoded=10.00]	1	.343
[bodysystemrecoded=11.00]	.	.
[Indicated dose=1]	.	.
[Indicated dose=2]	1	<.001
[Indicated dose=3]	.	.
[Sex=0]	1	.003
[Sex=1]	1	.647
[Sex=2]	1	.278
[Sex=3]	.	.
Age (months)	1	.003
Weight®	1	<.001
(Scale)		

Dependent Variable: mg/kg
Model: (Intercept), bodysystemrecoded, Indicated dose, Sex, Age (months), Weight®

a. Set to zero because this parameter is redundant.

b. Maximum likelihood estimate.

GGraph

Notes

Output Created		13-JUL-2022 17:28:53
Comments		
Input	Data	/Users/bonniepurcell/Desktop/SPSS /SPSS Whole data set correct sex.sav
	Active Dataset	DataSet1
	Filter	<none>
	Weight	<none>
	Split File	<none>
	N of Rows in Working Data File	2000
Syntax		GGRAPH<br>/GRAPHDATASET NAME="graphdataset" VARIABLES=mgkg CooksDistance MISSING=LISTWISE REPORTMISSING=NO<br>/GRAPHSPEC SOURCE=INLINE<br>/FITLINE TOTAL=NO SUBGROUP=NO.<br>BEGIN GPL<br>SOURCE: s=userSource(id("graphdataset"))<br>DATA: mgkg=col(source(s), name("mgkg"))<br>DATA: CooksDistance=col(source(s), name("CooksDistance"))<br>GUIDE: axis(dim(1), label("mg/kg"))<br>GUIDE: axis(dim(2), label("Cook's Distance"))<br>GUIDE: text.title(label("Scatter Plot of Cook's Distance by mg/kg"))<br>ELEMENT: point(position(mgkg*CooksDistance))<br>END GPL.
Resources	Processor Time	00:00:00.26
	Elapsed Time	00:00:00.00

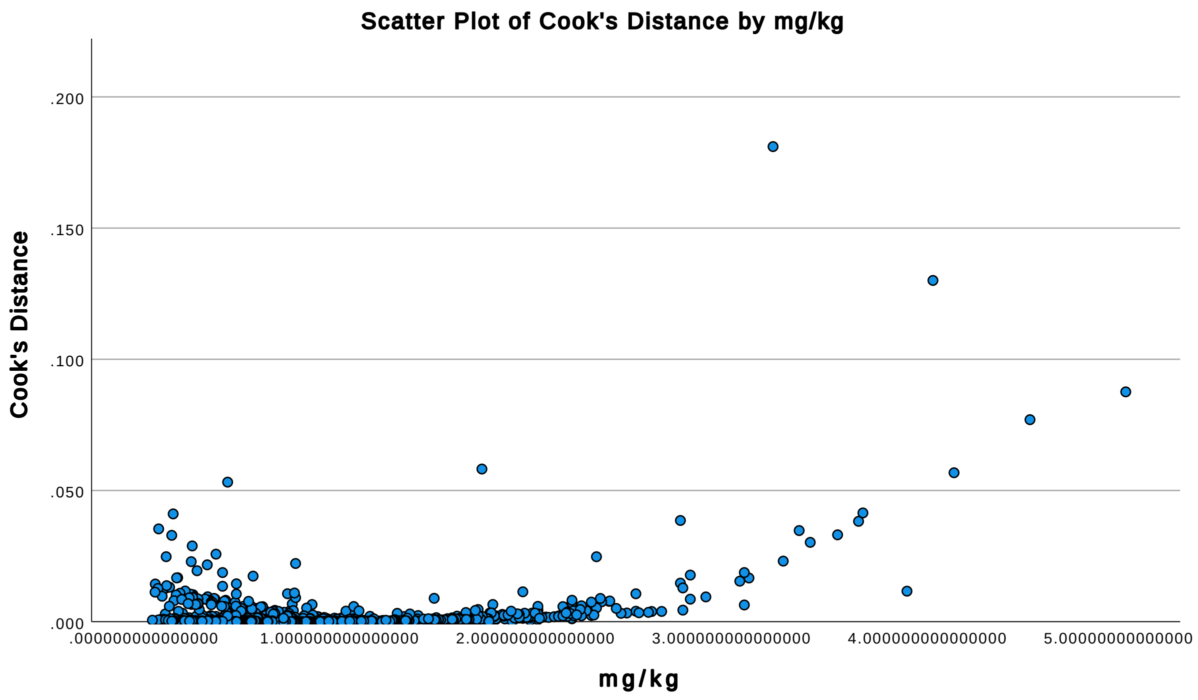

Generalized Linear Models

Notes

Output Created		13-JUL-2022 17:42:40
Comments		
Input	Data	/Users/bonniepurcell/Desktop/SPSS /SPSS Whole data set correct sex.sav
	Active Dataset	DataSet1
	Filter	CooksDistance <= 0.05 (FILTER)
	Weight	<none>
	Split File	<none>
	N of Rows in Working Data File	1991
Missing Value Handling	Definition of Missing	User-defined missing values for factor, subject and within-subject variables are treated as missing.
	Cases Used	Statistics are based on cases with valid data for all variables in the model.
Weight Handling		not applicable

Notes

Syntax		GENLIN mgkg BY bodysystemrecoded Indicateddose Sex (ORDER=ASCENDING) WITH Agemonths Weight®<br>/MODEL bodysystemrecoded Indicateddose Sex Agemonths Weight® INTERCEPT=YES<br>DISTRIBUTION=NORMAL LINK=IDENTITY<br>/CRITERIA SCALE=MLE COVB=MODEL PCONVERGE=1E-006 (ABSOLUTE) SINGULAR=1E-012 ANALYSISTYPE=3(LR)<br>CILEVEL=95 CITYPE=WALD LIKELIHOOD=FULL<br>/MISSING CLASSMISSING=EXCLUDE<br>/PRINT CPS DESCRIPTIVES MODELINFO FIT SUMMARY SOLUTION<br>/SAVE MEANPRED COOK RESID PEARSONRESID STDPEARSONRESID.
Resources	Processor Time	00:00:00.68
	Elapsed Time	00:00:01.00
Variables Created or Modified	Predicted Value of the Mean of the Response	MeanPredicted_2
	Raw Residual	Residual_2
	Pearson Residual	PearsonResidual_2
	Standardized Pearson Residual	StdPearsonResidual_2
	Cook's Distance	CooksDistance_1

Model Information

Dependent Variable	mg/kg
Probability Distribution	Normal
Link Function	Identity

Case Processing Summary

	N	Percent
Included	1879	94.4%
Excluded	112	5.6%
Total	1991	100.0%

Categorical Variable Information

			N	Percent
Factor	bodysystemrecoded	2.00	24	1.3%
		3.00	79	4.2%
		4.00	21	1.1%
		5.00	61	3.2%
		6.00	31	1.6%
		7.00	10	0.5%
		8.00	4	0.2%
		10.00	6	0.3%
		11.00	1643	87.4%
		Total	1879	100.0%
	Indicated dose	1	10	0.5%
		2	1793	95.4%
		3	76	4.0%
		Total	1879	100.0%
	Sex	0	113	6.0%
		1	734	39.1%
		2	203	10.8%
		3	829	44.1%
		Total	1879	100.0%

Continuous Variable Information

		N	Minimum	Maximum	Mean
Dependent Variable	mg/kg	1879	.032722513	3.88349515	.906831477
Covariate	Age (months)	1879	2	220	75.38
	Weight®	1879	1.56	90.00	19.3223

Continuous Variable Information

		Std. Deviation
Dependent Variable	mg/kg	.487697711
Covariate	Age (months)	49.433
	Weight®	12.57661

Goodness of Fit[a]

	Value	df	Value/df
Deviance	398.163	1864	.214
Scaled Deviance	1879.000	1864	
Pearson Chi-Square	398.163	1864	.214
Scaled Pearson Chi-Square	1879.000	1864	
Log Likelihood[b]	-1208.425		
Akaike's Information Criterion (AIC)	2448.851		
Finite Sample Corrected AIC (AICC)	2449.143		
Bayesian Information Criterion (BIC)	2537.467		
Consistent AIC (CAIC)	2553.467		

Dependent Variable: mg/kg
Model: (Intercept), bodysystemrecoded, Indicated dose, Sex, Age (months), Weight®[a]

a. Information criteria are in smaller-is-better form.

b. The full log likelihood function is displayed and used in computing information criteria.

Omnibus Test[a]

Likelihood Ratio Chi-Square	df	Sig.
216.052	14	.000

Dependent Variable: mg/kg
Model: (Intercept), bodysystemrecoded, Indicated dose, Sex, Age (months), Weight®[a]

a. Compares the fitted model against the intercept-only...

Tests of Model Effects

Source	Type III Likelihood Ratio Chi-Square	df	Sig.
(Intercept)	346.080	1	.000
bodysystemrecoded	32.941	7	<.001
Indicated dose	40.318	1	<.001
Sex	9.432	3	.024
Age (months)	7.808	1	.005
Weight®	30.802	1	<.001

Dependent Variable: mg/kg
Model: (Intercept), bodysystemrecoded, Indicated dose, Sex, Age (months), Weight®

Parameter Estimates

Parameter	B	Std. Error	95% Wald Confidence Interval		Hypothesis Test
			Lower	Upper	Wald Chi-Square
(Intercept)	1.458	.0772	1.306	1.609	356.981
[bodysystemrecoded=2.00]	.109	.0948	-.077	.295	1.326
[bodysystemrecoded=3.00]	.040	.0549	-.068	.147	.526
[bodysystemrecoded=4.00]	.132	.1019	-.068	.332	1.678
[bodysystemrecoded=5.00]	.291	.0638	.166	.416	20.752
[bodysystemrecoded=6.00]	.328	.1084	.115	.540	9.138
[bodysystemrecoded=7.00]	-1.020	.1627	-1.339	-.701	39.306
[bodysystemrecoded=8.00]	-.396	.2332	-.853	.061	2.889
[bodysystemrecoded=10.00]	.190	.1885	-.179	.560	1.020
[bodysystemrecoded=11.00]	0[a]	.	.	.	.
[Indicated dose=1]	0[a]	.	.	.	.
[Indicated dose=2]	-.461	.0722	-.602	-.319	40.754
[Indicated dose=3]	0[a]	.	.	.	.
[Sex=0]	.137	.0465	.046	.228	8.652
[Sex=1]	.013	.0234	-.033	.059	.294
[Sex=2]	.047	.0366	-.025	.118	1.627
[Sex=3]	0[a]	.	.	.	.
Age (months)	-.001	.0002	-.001	.000	7.824
Weight®	-.005	.0009	-.007	-.003	31.056
(Scale)	.212[b]	.0069	.199	.226	

Parameter Estimates

Parameter	Hypothesis Test	
	df	Sig.
(Intercept)	1	.000
[bodysystemrecoded=2.00]	1	.249
[bodysystemrecoded=3.00]	1	.468
[bodysystemrecoded=4.00]	1	.195
[bodysystemrecoded=5.00]	1	<.001
[bodysystemrecoded=6.00]	1	.003
[bodysystemrecoded=7.00]	1	<.001
[bodysystemrecoded=8.00]	1	.089
[bodysystemrecoded=10.00]	1	.313
[bodysystemrecoded=11.00]	.	.
[Indicated dose=1]	.	.
[Indicated dose=2]	1	<.001
[Indicated dose=3]	.	.
[Sex=0]	1	.003
[Sex=1]	1	.588
[Sex=2]	1	.202
[Sex=3]	.	.
Age (months)	1	.005
Weight®	1	<.001
(Scale)		

Dependent Variable: mg/kg
Model: (Intercept), bodysystemrecoded, Indicated dose, Sex, Age (months), Weight®

a. Set to zero because this parameter is redundant.

b. Maximum likelihood estimate.

GGraph

Notes

Output Created		13-JUL-2022 17:49:22
Comments		
Input	Data	/Users/bonniepurcell/Desktop/SPSS /SPSS Whole data set correct sex.sav
	Active Dataset	DataSet1
	Filter	CooksDistance <= 0.05 (FILTER)
	Weight	<none>
	Split File	<none>
	N of Rows in Working Data File	1991
Syntax		GGRAPH<br>/GRAPHDATASET NAME="graphdataset" VARIABLES=Residual_2 MISSING=LISTWISE REPORTMISSING=NO<br>/GRAPHSPEC SOURCE=INLINE.<br>BEGIN GPL<br>SOURCE: s=userSource(id("graphdataset"))<br>DATA: Residual_2=col(source(s), name("Residual_2"))<br>GUIDE: axis(dim(1), label("Raw Residual"))<br>GUIDE: axis(dim(2), label("Frequency"))<br>GUIDE: text.title(label("Simple Histogram of Raw Residual"))<br>ELEMENT: interval(position(summary.count(bin.rect(Residual_2))), shape.interior(shape.square))<br>END GPL.
Resources	Processor Time	00:00:00.35
	Elapsed Time	00:00:00.00

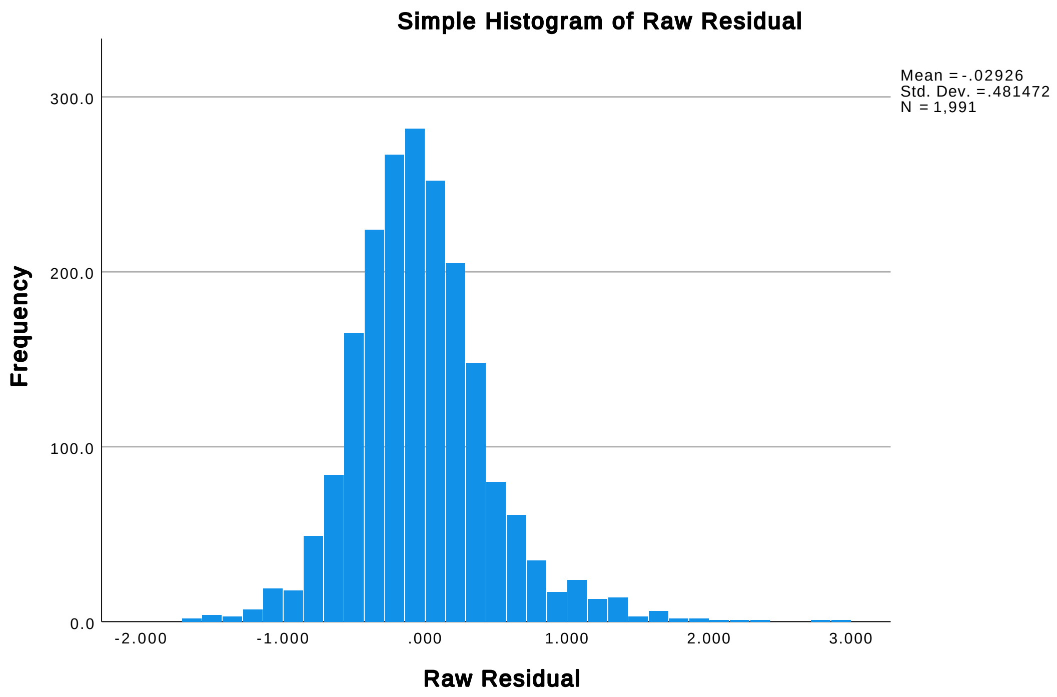

GGraph

Notes

Output Created		13-JUL-2022 17:50:17
Comments		
Input	Data	/Users/bonniepurcell/Desktop/SPSS /SPSS Whole data set correct sex.sav
	Active Dataset	DataSet1
	Filter	CooksDistance <= 0.05 (FILTER)
	Weight	<none>
	Split File	<none>
	N of Rows in Working Data File	1991

Notes

Syntax		GGRAPH<br>/GRAPHDATASET NAME="graphdataset" VARIABLES=mgkg MeanPredicted_2 MISSING=LISTWISE REPORTMISSING=NO<br>/GRAPHSPEC SOURCE=INLINE<br>/FITLINE TOTAL=NO SUBGROUP=NO.<br>BEGIN GPL<br>SOURCE: s=userSource (id("graphdataset"))<br>DATA: mgkg=col (source(s), name("mgkg"))<br>DATA: MeanPredicted_2=col (source(s), name ("MeanPredicted_2"))<br>GUIDE: axis(dim(1), label("mg/kg"))<br>GUIDE: axis(dim(2), label("Predicted Value of Mean of Response"))<br>GUIDE: text.title(label ("Scatter Plot of Predicted Value of Mean of Response by mg/kg"))<br>ELEMENT: point(position (mgkg*MeanPredicted_2))<br>END GPL.
Resources	Processor Time	00:00:00.22
	Elapsed Time	00:00:00.00

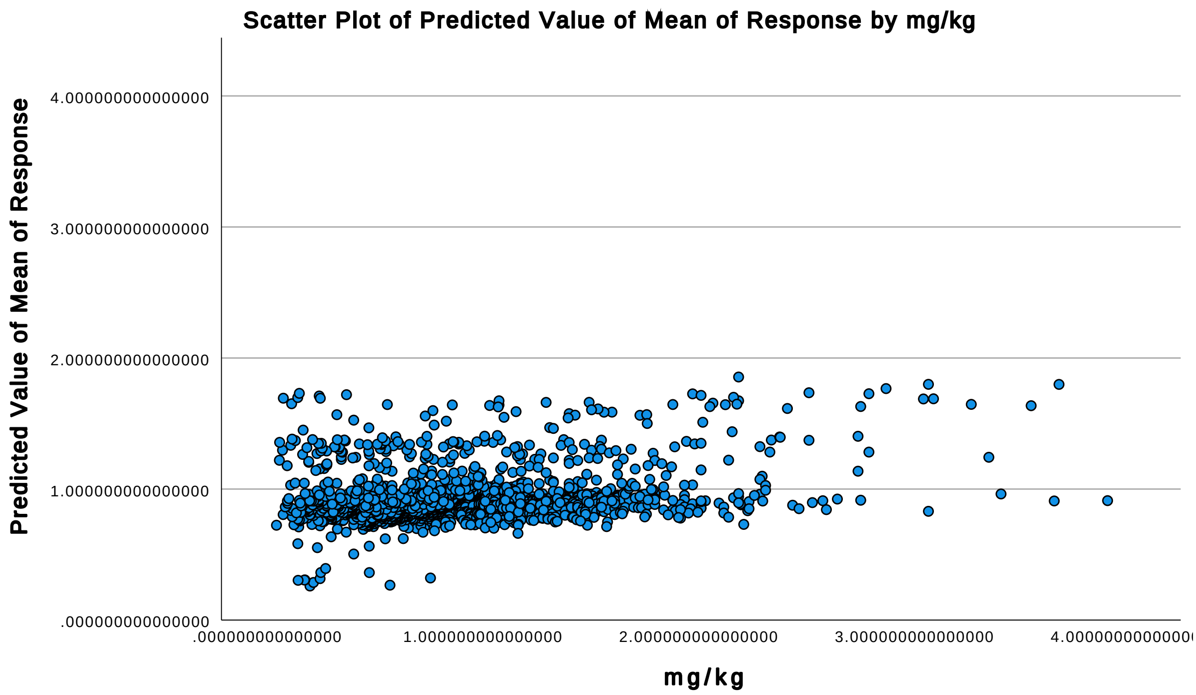

GGraph

Notes

Output Created		13-JUL-2022 17:51:38
Comments		
Input	Data	/Users/bonniepurcell/Desktop/SPSS /SPSS Whole data set correct sex.sav
	Active Dataset	DataSet1
	Filter	CooksDistance <= 0.05 (FILTER)
	Weight	<none>
	Split File	<none>
	N of Rows in Working Data File	1991
Syntax		GGRAPH<br>/GRAPHDATASET NAME="graphdataset" VARIABLES=mgkg CooksDistance_1 MISSING=LISTWISE REPORTMISSING=NO<br>/GRAPHSPEC SOURCE=INLINE<br>/FITLINE TOTAL=NO SUBGROUP=NO.<br>BEGIN GPL<br>SOURCE: s=userSource(id("graphdataset"))<br>DATA: mgkg=col(source(s), name("mgkg"))<br>DATA: CooksDistance_1=col(source(s), name("CooksDistance_1"))<br>GUIDE: axis(dim(1), label("mg/kg"))<br>GUIDE: axis(dim(2), label("Cook's Distance"))<br>GUIDE: text.title(label("Scatter Plot of Cook's Distance by mg/kg"))<br>ELEMENT: point(position(mgkg*CooksDistance_1))<br>END GPL.
Resources	Processor Time	00:00:00.24
	Elapsed Time	00:00:00.00

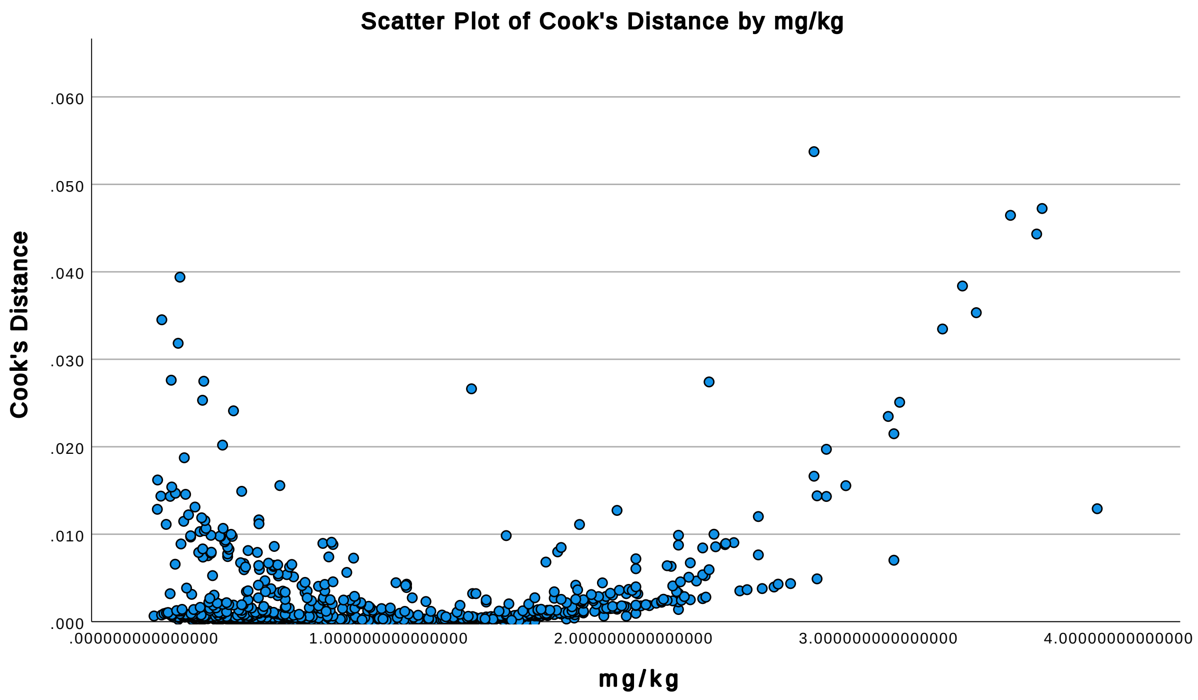

Generalized Linear Models

Notes

Output Created		15-JUL-2022 13:53:01
Comments		
Input	Data	/Users/bonniepurcell/Desktop/SPSS /SPSS Whole data set correct sex.sav
	Active Dataset	DataSet1
	Filter	CooksDistance <= 0.05 (FILTER)
	Weight	<none>
	Split File	<none>
	N of Rows in Working Data File	1991
Missing Value Handling	Definition of Missing	User-defined missing values for factor, subject and within-subject variables are treated as missing.
	Cases Used	Statistics are based on cases with valid data for all variables in the model.
Weight Handling		not applicable

Notes

Syntax		GENLIN mgkg BY Indicateddose bodysystemrecoded Sex (ORDER=ASCENDING) WITH Agemonths Weight®<br>/MODEL bodysystemrecoded Indicateddose Sex Agemonths Weight® INTERCEPT=YES<br>DISTRIBUTION=NORMAL LINK=IDENTITY<br>/CRITERIA SCALE=MLE COVB=MODEL PCONVERGE=1E-006 (ABSOLUTE) SINGULAR=1E-012 ANALYSISTYPE=3(LR)<br>CILEVEL=95 CITYPE=WALD LIKELIHOOD=FULL<br>/MISSING CLASSMISSING=EXCLUDE<br>/PRINT CPS DESCRIPTIVES MODELINFO FIT SUMMARY SOLUTION<br>/SAVE MEANPRED COOK RESID PEARSONRESID STDPEARSONRESID.
Resources	Processor Time	00:00:00.66
	Elapsed Time	00:00:00.00
Variables Created or Modified	Predicted Value of the Mean of the Response	MeanPredicted_3
	Raw Residual	Residual_3
	Pearson Residual	PearsonResidual_3
	Standardized Pearson Residual	StdPearsonResidual_3
	Cook's Distance	CooksDistance_2

Model Information

Dependent Variable	mg/kg
Probability Distribution	Normal
Link Function	Identity

Case Processing Summary

	N	Percent
Included	1879	94.4%
Excluded	112	5.6%
Total	1991	100.0%

Categorical Variable Information

			N	Percent
Factor	Indicated dose	1	10	0.5%
		2	1793	95.4%
		3	76	4.0%
		Total	1879	100.0%
	bodysystemrecoded	2.00	24	1.3%
		3.00	79	4.2%
		4.00	21	1.1%
		5.00	61	3.2%
		6.00	31	1.6%
		7.00	10	0.5%
		8.00	4	0.2%
		10.00	6	0.3%
		11.00	1643	87.4%
		Total	1879	100.0%
	Sex	0	113	6.0%
		1	734	39.1%
		2	203	10.8%
		3	829	44.1%
		Total	1879	100.0%

Continuous Variable Information

		N	Minimum	Maximum	Mean
Dependent Variable	mg/kg	1879	.032722513	3.88349515	.906831477
Covariate	Age (months)	1879	2	220	75.38
	Weight®	1879	1.56	90.00	19.3223

Continuous Variable Information

		Std. Deviation
Dependent Variable	mg/kg	.487697711
Covariate	Age (months)	49.433
	Weight®	12.57661

Goodness of Fit[a]

	Value	df	Value/df
Deviance	398.163	1864	.214
Scaled Deviance	1879.000	1864	
Pearson Chi-Square	398.163	1864	.214
Scaled Pearson Chi-Square	1879.000	1864	
Log Likelihood[b]	-1208.425		
Akaike's Information Criterion (AIC)	2448.851		
Finite Sample Corrected AIC (AICC)	2449.143		
Bayesian Information Criterion (BIC)	2537.467		
Consistent AIC (CAIC)	2553.467		

Dependent Variable: mg/kg
Model: (Intercept), bodysystemrecoded, Indicated dose, Sex, Age (months), Weight®[a]

a. Information criteria are in smaller-is-better form.

b. The full log likelihood function is displayed and used in computing information criteria.

Omnibus Test[a]

Likelihood Ratio Chi-Square	df	Sig.
216.052	14	.000

Dependent Variable: mg/kg
Model: (Intercept), bodysystemrecoded, Indicated dose, Sex, Age (months), Weight®[a]

a. Compares the fitted model against the intercept-only...

Tests of Model Effects

Source	Type III Likelihood Ratio Chi-Square	df	Sig.
(Intercept)	346.080	1	.000
bodysystemrecoded	32.941	7	<.001
Indicated dose	40.318	1	<.001
Sex	9.432	3	.024
Age (months)	7.808	1	.005
Weight®	30.802	1	<.001

Dependent Variable: mg/kg
Model: (Intercept), bodysystemrecoded, Indicated dose, Sex, Age (months), Weight®

Parameter Estimates

Parameter	B	Std. Error	95% Wald Confidence Interval		Hypothesis Test
			Lower	Upper	Wald Chi-Square
(Intercept)	1.458	.0772	1.306	1.609	356.981
[bodysystemrecoded=2.00]	.109	.0948	-.077	.295	1.326
[bodysystemrecoded=3.00]	.040	.0549	-.068	.147	.526
[bodysystemrecoded=4.00]	.132	.1019	-.068	.332	1.678
[bodysystemrecoded=5.00]	.291	.0638	.166	.416	20.752
[bodysystemrecoded=6.00]	.328	.1084	.115	.540	9.138
[bodysystemrecoded=7.00]	-1.020	.1627	-1.339	-.701	39.306
[bodysystemrecoded=8.00]	-.396	.2332	-.853	.061	2.889
[bodysystemrecoded=10.00]	.190	.1885	-.179	.560	1.020
[bodysystemrecoded=11.00]	0[a]	.	.	.	.
[Indicated dose=1]	0[a]	.	.	.	.
[Indicated dose=2]	-.461	.0722	-.602	-.319	40.754
[Indicated dose=3]	0[a]	.	.	.	.
[Sex=0]	.137	.0465	.046	.228	8.652
[Sex=1]	.013	.0234	-.033	.059	.294
[Sex=2]	.047	.0366	-.025	.118	1.627
[Sex=3]	0[a]	.	.	.	.
Age (months)	-.001	.0002	-.001	.000	7.824
Weight®	-.005	.0009	-.007	-.003	31.056
(Scale)	.212[b]	.0069	.199	.226	

Parameter Estimates

Parameter	Hypothesis Test df	Sig.
(Intercept)	1	.000
[bodysystemrecoded=2.00]	1	.249
[bodysystemrecoded=3.00]	1	.468
[bodysystemrecoded=4.00]	1	.195
[bodysystemrecoded=5.00]	1	<.001
[bodysystemrecoded=6.00]	1	.003
[bodysystemrecoded=7.00]	1	<.001
[bodysystemrecoded=8.00]	1	.089
[bodysystemrecoded=10.00]	1	.313
[bodysystemrecoded=11.00]	.	.
[Indicated dose=1]	.	.
[Indicated dose=2]	1	<.001
[Indicated dose=3]	.	.
[Sex=0]	1	.003
[Sex=1]	1	.588
[Sex=2]	1	.202
[Sex=3]	.	.
Age (months)	1	.005
Weight®	1	<.001
(Scale)		

Dependent Variable: mg/kg
Model: (Intercept), bodysystemrecoded, Indicated dose, Sex, Age (months), Weight®

a. Set to zero because this parameter is redundant.

b. Maximum likelihood estimate.

Generalized Linear Models

Notes

Output Created		15-JUL-2022 16:33:41
Comments		
Input	Data	/Users/bonniepurcell/Desktop/SPSS /SPSS Whole data set correct sex.sav
	Active Dataset	DataSet1
	Filter	CooksDistance <= 0.05 (FILTER)
	Weight	<none>
	Split File	<none>
	N of Rows in Working Data File	1991
Missing Value Handling	Definition of Missing	User-defined missing values for factor, subject and within-subject variables are treated as missing.
	Cases Used	Statistics are based on cases with valid data for all variables in the model.
Weight Handling		not applicable
Syntax		GENLIN mgkg BY Indicateddose Sex bodysystemrecoded (ORDER=ASCENDING) WITH Agemonths Weight®<br>/MODEL bodysystemrecoded Indicateddose Sex Agemonths Weight® INTERCEPT=YES<br>DISTRIBUTION=NORMAL LINK=IDENTITY<br>/CRITERIA SCALE=MLE COVB=MODEL PCONVERGE=1E-006 (ABSOLUTE) SINGULAR=1E-012 ANALYSISTYPE=3(LR)<br>CILEVEL=95 CITYPE=WALD LIKELIHOOD=FULL<br>/MISSING CLASSMISSING=EXCLUDE<br>/PRINT CPS DESCRIPTIVES MODELINFO FIT SUMMARY SOLUTION.
Resources	Processor Time	00:00:00.64
	Elapsed Time	00:00:01.00

Model Information

Dependent Variable	mg/kg
Probability Distribution	Normal
Link Function	Identity

Case Processing Summary

	N	Percent
Included	1879	94.4%
Excluded	112	5.6%
Total	1991	100.0%

Categorical Variable Information

			N	Percent
Factor	Indicated dose	1	10	0.5%
		2	1793	95.4%
		3	76	4.0%
		Total	1879	100.0%
	Sex	0	113	6.0%
		1	734	39.1%
		2	203	10.8%
		3	829	44.1%
		Total	1879	100.0%
	bodysystemrecoded	2.00	24	1.3%
		3.00	79	4.2%
		4.00	21	1.1%
		5.00	61	3.2%
		6.00	31	1.6%
		7.00	10	0.5%
		8.00	4	0.2%
		10.00	6	0.3%
		11.00	1643	87.4%
		Total	1879	100.0%

Continuous Variable Information

		N	Minimum	Maximum	Mean
Dependent Variable	mg/kg	1879	.032722513	3.88349515	.906831477
Covariate	Age (months)	1879	2	220	75.38
	Weight®	1879	1.56	90.00	19.3223

Continuous Variable Information

		Std. Deviation
Dependent Variable	mg/kg	.487697711
Covariate	Age (months)	49.433
	Weight®	12.57661

Goodness of Fit[a]

	Value	df	Value/df
Deviance	398.163	1864	.214
Scaled Deviance	1879.000	1864	
Pearson Chi-Square	398.163	1864	.214
Scaled Pearson Chi-Square	1879.000	1864	
Log Likelihood[b]	-1208.425		
Akaike's Information Criterion (AIC)	2448.851		
Finite Sample Corrected AIC (AICC)	2449.143		
Bayesian Information Criterion (BIC)	2537.467		
Consistent AIC (CAIC)	2553.467		

Dependent Variable: mg/kg
Model: (Intercept), bodysystemrecoded, Indicated dose, Sex, Age (months), Weight®[a]

a. Information criteria are in smaller-is-better form.

b. The full log likelihood function is displayed and used in computing information criteria.

Omnibus Test[a]

Likelihood Ratio Chi-Square	df	Sig.
216.052	14	.000

Dependent Variable: mg/kg
Model: (Intercept), bodysystemrecoded, Indicated dose, Sex, Age (months), Weight®[a]

a. Compares the fitted model against the intercept-only...

Tests of Model Effects

	Type III		
Source	Likelihood Ratio Chi-Square	df	Sig.
(Intercept)	346.080	1	.000
bodysystemrecoded	32.941	7	<.001
Indicated dose	40.318	1	<.001
Sex	9.432	3	.024
Age (months)	7.808	1	.005
Weight®	30.802	1	<.001

Dependent Variable: mg/kg
Model: (Intercept), bodysystemrecoded, Indicated dose, Sex, Age (months), Weight®

Parameter Estimates

Parameter	B	Std. Error	95% Wald Confidence Interval		Hypothesis Test
			Lower	Upper	Wald Chi-Square
(Intercept)	1.458	.0772	1.306	1.609	356.981
[bodysystemrecoded=2.00]	.109	.0948	-.077	.295	1.326
[bodysystemrecoded=3.00]	.040	.0549	-.068	.147	.526
[bodysystemrecoded=4.00]	.132	.1019	-.068	.332	1.678
[bodysystemrecoded=5.00]	.291	.0638	.166	.416	20.752
[bodysystemrecoded=6.00]	.328	.1084	.115	.540	9.138
[bodysystemrecoded=7.00]	-1.020	.1627	-1.339	-.701	39.306
[bodysystemrecoded=8.00]	-.396	.2332	-.853	.061	2.889
[bodysystemrecoded=10.00]	.190	.1885	-.179	.560	1.020
[bodysystemrecoded=11.00]	0[a]	.	.	.	.
[Indicated dose=1]	0[a]	.	.	.	.
[Indicated dose=2]	-.461	.0722	-.602	-.319	40.754
[Indicated dose=3]	0[a]	.	.	.	.
[Sex=0]	.137	.0465	.046	.228	8.652
[Sex=1]	.013	.0234	-.033	.059	.294
[Sex=2]	.047	.0366	-.025	.118	1.627
[Sex=3]	0[a]	.	.	.	.
Age (months)	-.001	.0002	-.001	.000	7.824
Weight®	-.005	.0009	-.007	-.003	31.056
(Scale)	.212[b]	.0069	.199	.226	

Parameter Estimates

Parameter	Hypothesis Test	
	df	Sig.
(Intercept)	1	.000
[bodysystemrecoded=2.00]	1	.249
[bodysystemrecoded=3.00]	1	.468
[bodysystemrecoded=4.00]	1	.195
[bodysystemrecoded=5.00]	1	<.001
[bodysystemrecoded=6.00]	1	.003
[bodysystemrecoded=7.00]	1	<.001
[bodysystemrecoded=8.00]	1	.089
[bodysystemrecoded=10.00]	1	.313
[bodysystemrecoded=11.00]	.	.
[Indicated dose=1]	.	.
[Indicated dose=2]	1	<.001
[Indicated dose=3]	.	.
[Sex=0]	1	.003
[Sex=1]	1	.588
[Sex=2]	1	.202
[Sex=3]	.	.
Age (months)	1	.005
Weight®	1	<.001
(Scale)		

Dependent Variable: mg/kg
Model: (Intercept), bodysystemrecoded, Indicated dose, Sex, Age (months), Weight®

a. Set to zero because this parameter is redundant.

b. Maximum likelihood estimate.

Generalized Linear Models

Notes

Output Created		15-JUL-2022 16:34:44
Comments		
Input	Data	/Users/bonniepurcell/Desktop/SPSS /SPSS Whole data set correct sex.sav
	Active Dataset	DataSet1
	Filter	CooksDistance <= 0.05 (FILTER)
	Weight	<none>
	Split File	<none>
	N of Rows in Working Data File	1991
Missing Value Handling	Definition of Missing	User-defined missing values for factor, subject and within-subject variables are treated as missing.
	Cases Used	Statistics are based on cases with valid data for all variables in the model.
Weight Handling		not applicable
Syntax		GENLIN mgkg BY Indicateddose Sex bodysystemrecoded (ORDER=DATA) WITH Agemonths Weight®<br>/MODEL bodysystemrecoded Indicateddose Sex Agemonths Weight® INTERCEPT=YES<br>DISTRIBUTION=NORMAL LINK=IDENTITY<br>/CRITERIA SCALE=MLE COVB=MODEL PCONVERGE=1E-006 (ABSOLUTE) SINGULAR=1E-012 ANALYSISTYPE=3(LR)<br>CILEVEL=95 CITYPE=WALD LIKELIHOOD=FULL<br>/MISSING CLASSMISSING=EXCLUDE<br>/PRINT CPS DESCRIPTIVES MODELINFO FIT SUMMARY SOLUTION.
Resources	Processor Time	00:00:00.58
	Elapsed Time	00:00:01.00

Model Information

Dependent Variable	mg/kg
Probability Distribution	Normal
Link Function	Identity

Case Processing Summary

	N	Percent
Included	1879	94.4%
Excluded	112	5.6%
Total	1991	100.0%

Categorical Variable Information

			N	Percent
Factor	Indicated dose	2	1793	95.4%
		3	76	4.0%
		1	10	0.5%
		Total	1879	100.0%
	Sex	3	829	44.1%
		0	113	6.0%
		1	734	39.1%
		2	203	10.8%
		Total	1879	100.0%
	bodysystemrecoded	11.00	1643	87.4%
		6.00	31	1.6%
		3.00	79	4.2%
		2.00	24	1.3%
		5.00	61	3.2%
		10.00	6	0.3%
		8.00	4	0.2%
		4.00	21	1.1%
		7.00	10	0.5%
		Total	1879	100.0%

Continuous Variable Information

		N	Minimum	Maximum	Mean
Dependent Variable	mg/kg	1879	.032722513	3.88349515	.906831477
Covariate	Age (months)	1879	2	220	75.38
	Weight®	1879	1.56	90.00	19.3223

Continuous Variable Information

		Std. Deviation
Dependent Variable	mg/kg	.487697711
Covariate	Age (months)	49.433
	Weight®	12.57661

Goodness of Fit[a]

	Value	df	Value/df
Deviance	398.163	1864	.214
Scaled Deviance	1879.000	1864	
Pearson Chi-Square	398.163	1864	.214
Scaled Pearson Chi-Square	1879.000	1864	
Log Likelihood[b]	-1208.425		
Akaike's Information Criterion (AIC)	2448.851		
Finite Sample Corrected AIC (AICC)	2449.143		
Bayesian Information Criterion (BIC)	2537.467		
Consistent AIC (CAIC)	2553.467		

Dependent Variable: mg/kg
Model: (Intercept), bodysystemrecoded, Indicated dose, Sex, Age (months), Weight®[a]

a. Information criteria are in smaller-is-better form.

b. The full log likelihood function is displayed and used in computing information criteria.

Omnibus Test[a]

Likelihood Ratio Chi-Square	df	Sig.
216.052	14	.000

Dependent Variable: mg/kg
Model: (Intercept), bodysystemrecoded, Indicated dose, Sex, Age (months), Weight®[a]

a. Compares the fitted model against the intercept-only...

Tests of Model Effects

	Type III		
Source	Likelihood Ratio Chi-Square	df	Sig.
(Intercept)	346.080	1	.000
bodysystemrecoded	32.941	7	<.001
Indicated dose	40.318	1	<.001
Sex	9.432	3	.024
Age (months)	7.808	1	.005
Weight®	30.802	1	<.001

Dependent Variable: mg/kg
Model: (Intercept), bodysystemrecoded, Indicated dose, Sex, Age (months), Weight®

Parameter Estimates

Parameter	B	Std. Error	95% Wald Confidence Interval		Hypothesis Test
			Lower	Upper	Wald Chi-Square
(Intercept)	.484	.1525	.185	.783	10.087
[bodysystemrecoded=11.00]	1.020	.1627	.701	1.339	39.306
[bodysystemrecoded=6.00]	1.348	.1676	1.019	1.676	64.676
[bodysystemrecoded=3.00]	1.060	.1706	.726	1.394	38.592
[bodysystemrecoded=2.00]	1.129	.1879	.761	1.497	36.137
[bodysystemrecoded=5.00]	1.311	.1656	.986	1.635	62.679
[bodysystemrecoded=10.00]	1.210	.2491	.722	1.699	23.622
[bodysystemrecoded=8.00]	.624	.2748	.085	1.162	5.154
[bodysystemrecoded=4.00]	1.152	.1911	.777	1.527	36.337
[bodysystemrecoded=7.00]	0[a]	.	.	.	.
[Indicated dose=2]	-.461	.0722	-.602	-.319	40.754
[Indicated dose=3]	0[a]	.	.	.	.
[Indicated dose=1]	0[a]	.	.	.	.
[Sex=3]	-.047	.0366	-.118	.025	1.627
[Sex=0]	.090	.0543	-.016	.197	2.756
[Sex=1]	-.034	.0371	-.107	.039	.839
[Sex=2]	0[a]	.	.	.	.
Age (months)	-.001	.0002	-.001	.000	7.824
Weight®	-.005	.0009	-.007	-.003	31.056
(Scale)	.212[b]	.0069	.199	.226	

Parameter Estimates

Parameter	Hypothesis Test df	Sig.
(Intercept)	1	.001
[bodysystemrecoded=11.00]	1	<.001
[bodysystemrecoded=6.00]	1	<.001
[bodysystemrecoded=3.00]	1	<.001
[bodysystemrecoded=2.00]	1	<.001
[bodysystemrecoded=5.00]	1	<.001
[bodysystemrecoded=10.00]	1	<.001
[bodysystemrecoded=8.00]	1	.023
[bodysystemrecoded=4.00]	1	<.001
[bodysystemrecoded=7.00]	.	.
[Indicated dose=2]	1	<.001
[Indicated dose=3]	.	.
[Indicated dose=1]	.	.
[Sex=3]	1	.202
[Sex=0]	1	.097
[Sex=1]	1	.360
[Sex=2]	.	.
Age (months)	1	.005
Weight®	1	<.001
(Scale)		

Dependent Variable: mg/kg
Model: (Intercept), bodysystemrecoded, Indicated dose, Sex, Age (months), Weight®

a. Set to zero because this parameter is redundant.

b. Maximum likelihood estimate.

Generalized Linear Models

Notes

Output Created		15-JUL-2022 16:35:57
Comments		
Input	Data	/Users/bonniepurcell/Desktop/SPSS /SPSS Whole data set correct sex.sav
	Active Dataset	DataSet1
	Filter	CooksDistance <= 0.05 (FILTER)
	Weight	<none>
	Split File	<none>
	N of Rows in Working Data File	1991
Missing Value Handling	Definition of Missing	User-defined missing values for factor, subject and within-subject variables are treated as missing.
	Cases Used	Statistics are based on cases with valid data for all variables in the model.
Weight Handling		not applicable
Syntax		GENLIN mgkg BY Indicateddose Sex bodysystemrecoded (ORDER=ASCENDING) WITH Agemonths Weight® /MODEL bodysystemrecoded Indicateddose Sex Agemonths Weight® INTERCEPT=YES DISTRIBUTION=NORMAL LINK=IDENTITY /CRITERIA SCALE=MLE COVB=MODEL PCONVERGE=1E-006 (ABSOLUTE) SINGULAR=1E-012 ANALYSISTYPE=3(LR) CILEVEL=95 CITYPE=WALD LIKELIHOOD=FULL /MISSING CLASSMISSING=EXCLUDE /PRINT CPS DESCRIPTIVES MODELINFO FIT SUMMARY SOLUTION.
Resources	Processor Time	00:00:00.63
	Elapsed Time	00:00:01.00

Model Information

Dependent Variable	mg/kg
Probability Distribution	Normal
Link Function	Identity

Case Processing Summary

	N	Percent
Included	1879	94.4%
Excluded	112	5.6%
Total	1991	100.0%

Categorical Variable Information

			N	Percent
Factor	Indicated dose	1	10	0.5%
		2	1793	95.4%
		3	76	4.0%
		Total	1879	100.0%
	Sex	0	113	6.0%
		1	734	39.1%
		2	203	10.8%
		3	829	44.1%
		Total	1879	100.0%
	bodysystemrecoded	2.00	24	1.3%
		3.00	79	4.2%
		4.00	21	1.1%
		5.00	61	3.2%
		6.00	31	1.6%
		7.00	10	0.5%
		8.00	4	0.2%
		10.00	6	0.3%
		11.00	1643	87.4%
		Total	1879	100.0%

Continuous Variable Information

		N	Minimum	Maximum	Mean
Dependent Variable	mg/kg	1879	.032722513	3.88349515	.906831477
Covariate	Age (months)	1879	2	220	75.38
	Weight®	1879	1.56	90.00	19.3223

Continuous Variable Information

		Std. Deviation
Dependent Variable	mg/kg	.487697711
Covariate	Age (months)	49.433
	Weight®	12.57661

Goodness of Fit[a]

	Value	df	Value/df
Deviance	398.163	1864	.214
Scaled Deviance	1879.000	1864	
Pearson Chi-Square	398.163	1864	.214
Scaled Pearson Chi-Square	1879.000	1864	
Log Likelihood[b]	-1208.425		
Akaike's Information Criterion (AIC)	2448.851		
Finite Sample Corrected AIC (AICC)	2449.143		
Bayesian Information Criterion (BIC)	2537.467		
Consistent AIC (CAIC)	2553.467		

Dependent Variable: mg/kg
Model: (Intercept), bodysystemrecoded, Indicated dose, Sex, Age (months), Weight®[a]

a. Information criteria are in smaller-is-better form.

b. The full log likelihood function is displayed and used in computing information criteria.

Omnibus Test[a]

Likelihood Ratio Chi-Square	df	Sig.
216.052	14	.000

Dependent Variable: mg/kg
Model: (Intercept), bodysystemrecoded, Indicated dose, Sex, Age (months), Weight®[a]

a. Compares the fitted model against the intercept-only...

Tests of Model Effects

Source	Type III Likelihood Ratio Chi-Square	df	Sig.
(Intercept)	346.080	1	.000
bodysystemrecoded	32.941	7	<.001
Indicated dose	40.318	1	<.001
Sex	9.432	3	.024
Age (months)	7.808	1	.005
Weight®	30.802	1	<.001

Dependent Variable: mg/kg
Model: (Intercept), bodysystemrecoded, Indicated dose, Sex, Age (months), Weight®

Parameter Estimates

Parameter	B	Std. Error	95% Wald Confidence Interval		Hypothesis Test
			Lower	Upper	Wald Chi-Square
(Intercept)	1.458	.0772	1.306	1.609	356.981
[bodysystemrecoded=2.00]	.109	.0948	-.077	.295	1.326
[bodysystemrecoded=3.00]	.040	.0549	-.068	.147	.526
[bodysystemrecoded=4.00]	.132	.1019	-.068	.332	1.678
[bodysystemrecoded=5.00]	.291	.0638	.166	.416	20.752
[bodysystemrecoded=6.00]	.328	.1084	.115	.540	9.138
[bodysystemrecoded=7.00]	-1.020	.1627	-1.339	-.701	39.306
[bodysystemrecoded=8.00]	-.396	.2332	-.853	.061	2.889
[bodysystemrecoded=10.00]	.190	.1885	-.179	.560	1.020
[bodysystemrecoded=11.00]	0[a]	.	.	.	.
[Indicated dose=1]	0[a]	.	.	.	.
[Indicated dose=2]	-.461	.0722	-.602	-.319	40.754
[Indicated dose=3]	0[a]	.	.	.	.
[Sex=0]	.137	.0465	.046	.228	8.652
[Sex=1]	.013	.0234	-.033	.059	.294
[Sex=2]	.047	.0366	-.025	.118	1.627
[Sex=3]	0[a]	.	.	.	.
Age (months)	-.001	.0002	-.001	.000	7.824
Weight®	-.005	.0009	-.007	-.003	31.056
(Scale)	.212[b]	.0069	.199	.226	

Parameter Estimates

Parameter	Hypothesis Test df	Sig.
(Intercept)	1	.000
[bodysystemrecoded=2.00]	1	.249
[bodysystemrecoded=3.00]	1	.468
[bodysystemrecoded=4.00]	1	.195
[bodysystemrecoded=5.00]	1	<.001
[bodysystemrecoded=6.00]	1	.003
[bodysystemrecoded=7.00]	1	<.001
[bodysystemrecoded=8.00]	1	.089
[bodysystemrecoded=10.00]	1	.313
[bodysystemrecoded=11.00]	.	.
[Indicated dose=1]	.	.
[Indicated dose=2]	1	<.001
[Indicated dose=3]	.	.
[Sex=0]	1	.003
[Sex=1]	1	.588
[Sex=2]	1	.202
[Sex=3]	.	.
Age (months)	1	.005
Weight®	1	<.001
(Scale)		

Dependent Variable: mg/kg
Model: (Intercept), bodysystemrecoded, Indicated dose, Sex, Age (months), Weight®

a. Set to zero because this parameter is redundant.

b. Maximum likelihood estimate.

Regression

Notes

Output Created		15-JUL-2022 17:02:45
Comments		
Input	Data	/Users/bonniepurcell/Desktop/SPSS /SPSS Whole data set correct sex.sav
	Active Dataset	DataSet1
	Filter	CooksDistance <= 0.05 (FILTER)
	Weight	<none>
	Split File	<none>
	N of Rows in Working Data File	1991
Missing Value Handling	Definition of Missing	User-defined missing values are treated as missing.
	Cases Used	Statistics are based on cases with no missing values for any variable used.
Syntax		REGRESSION /MISSING LISTWISE /STATISTICS COEFF OUTS CI(95) R ANOVA CHANGE /CRITERIA=PIN(.05) POUT(.10) /NOORIGIN /DEPENDENT mgkg /METHOD=ENTER Agemonths Weight® Indicateddose Sex bodysystemrecoded.
Resources	Processor Time	00:00:00.06
	Elapsed Time	00:00:00.00
	Memory Required	7968 bytes
	Additional Memory Required for Residual Plots	0 bytes

Variables Entered/Removed[a]

Model	Variables Entered	Variables Removed	Method
1	bodysystemrecoded, Sex, Weight®, Indicated dose, Age (months)[b]	.	Enter

a. Dependent Variable: mg/kg

b. All requested variables entered.

Model Summary

Model	R	R Square	Adjusted R Square	Std. Error of the Estimate	Change Statistics	
					R Square Change	F Change
1	.309[a]	.095	.093	.464476551	.095	39.494

Model Summary

Model	Change Statistics		
	df1	df2	Sig. F Change
1	5	1873	<.001

a. Predictors: (Constant), bodysystemrecoded, Sex, Weight®, Indicated dose, Age (months)

ANOVA[a]

Model		Sum of Squares	df	Mean Square	F	Sig.
1	Regression	42.602	5	8.520	39.494	<.001[b]
	Residual	404.078	1873	.216		
	Total	446.681	1878			

a. Dependent Variable: mg/kg

b. Predictors: (Constant), bodysystemrecoded, Sex, Weight®, Indicated dose, Age (months)

Coefficients[a]

Model		Unstandardized Coefficients		Standardized Coefficients	t	Sig.
		B	Std. Error	Beta		
1	(Constant)	.053	.129		.408	.683
	Age (months)	-.001	.000	-.073	-3.124	.002
	Weight®	-.005	.001	-.120	-5.327	<.001
	Indicated dose	.593	.052	.257	11.488	<.001
	Sex	-.019	.010	-.041	-1.841	.066
	bodysystemrecoded	-.017	.005	-.080	-3.415	<.001

Coefficients[a]

Model		95.0% Confidence Interval for B	
		Lower Bound	Upper Bound
1	(Constant)	-.201	.306
	Age (months)	-.001	.000
	Weight®	-.006	-.003
	Indicated dose	.492	.695
	Sex	-.039	.001
	bodysystemrecoded	-.027	-.007

a. Dependent Variable: mg/kg

Generalized Linear Models

Notes

Output Created		15-JUL-2022 17:06:38
Comments		
Input	Data	/Users/bonniepurcell/Desktop/SPSS /SPSS Whole data set correct sex.sav
	Active Dataset	DataSet1
	Filter	CooksDistance <= 0.05 (FILTER)
	Weight	<none>
	Split File	<none>
	N of Rows in Working Data File	1991
Missing Value Handling	Definition of Missing	User-defined missing values for factor, subject and within-subject variables are treated as missing.
	Cases Used	Statistics are based on cases with valid data for all variables in the model.
Weight Handling		not applicable
Syntax		GENLIN mgkg BY Indicateddose Sex bodysystemrecoded (ORDER=ASCENDING) WITH Agemonths Weight® /MODEL bodysystemrecoded Indicateddose Sex Agemonths Weight® INTERCEPT=YES DISTRIBUTION=NORMAL LINK=IDENTITY /CRITERIA SCALE=MLE COVB=MODEL PCONVERGE=1E-006 (ABSOLUTE) SINGULAR=1E-012 ANALYSISTYPE=3(LR) CILEVEL=95 CITYPE=WALD LIKELIHOOD=FULL /MISSING CLASSMISSING=EXCLUDE /PRINT CPS DESCRIPTIVES MODELINFO FIT SUMMARY SOLUTION GEF /SAVE MEANPRED COOK RESID PEARSONRESID STDPEARSONRESID.
Resources	Processor Time	00:00:00.68
	Elapsed Time	00:00:01.00

Notes

Variables Created or Modified	Predicted Value of the Mean of the Response	MeanPredicted_4
	Raw Residual	Residual_4
	Pearson Residual	PearsonResidual_4
	Standardized Pearson Residual	StdPearsonResidual_4
	Cook's Distance	CooksDistance_3

Model Information

Dependent Variable	mg/kg
Probability Distribution	Normal
Link Function	Identity

Case Processing Summary

	N	Percent
Included	1879	94.4%
Excluded	112	5.6%
Total	1991	100.0%

Categorical Variable Information

			N	Percent
Factor	Indicated dose	1	10	0.5%
		2	1793	95.4%
		3	76	4.0%
		Total	1879	100.0%
	Sex	0	113	6.0%
		1	734	39.1%
		2	203	10.8%
		3	829	44.1%
		Total	1879	100.0%
	bodysystemrecoded	2.00	24	1.3%
		3.00	79	4.2%
		4.00	21	1.1%
		5.00	61	3.2%
		6.00	31	1.6%
		7.00	10	0.5%
		8.00	4	0.2%
		10.00	6	0.3%
		11.00	1643	87.4%
		Total	1879	100.0%

Continuous Variable Information

		N	Minimum	Maximum	Mean
Dependent Variable	mg/kg	1879	.032722513	3.88349515	.906831477
Covariate	Age (months)	1879	2	220	75.38
	Weight®	1879	1.56	90.00	19.3223

Continuous Variable Information

		Std. Deviation
Dependent Variable	mg/kg	.487697711
Covariate	Age (months)	49.433
	Weight®	12.57661

Goodness of Fit[a]

	Value	df	Value/df
Deviance	398.163	1864	.214
Scaled Deviance	1879.000	1864	
Pearson Chi-Square	398.163	1864	.214
Scaled Pearson Chi-Square	1879.000	1864	
Log Likelihood[b]	-1208.425		
Akaike's Information Criterion (AIC)	2448.851		
Finite Sample Corrected AIC (AICC)	2449.143		
Bayesian Information Criterion (BIC)	2537.467		
Consistent AIC (CAIC)	2553.467		

Dependent Variable: mg/kg
Model: (Intercept), bodysystemrecoded, Indicated dose, Sex, Age (months), Weight®[a]

a. Information criteria are in smaller-is-better form.

b. The full log likelihood function is displayed and used in computing information criteria.

Omnibus Test[a]

Likelihood Ratio Chi-Square	df	Sig.
216.052	14	.000

Dependent Variable: mg/kg
Model: (Intercept), bodysystemrecoded, Indicated dose, Sex, Age (months), Weight®[a]

a. Compares the fitted model against the intercept-only...

Tests of Model Effects

Source	Type III Likelihood Ratio Chi-Square	df	Sig.
(Intercept)	346.080	1	.000
bodysystemrecoded	32.941	7	<.001
Indicated dose	40.318	1	<.001
Sex	9.432	3	.024
Age (months)	7.808	1	.005
Weight®	30.802	1	<.001

Dependent Variable: mg/kg
Model: (Intercept), bodysystemrecoded, Indicated dose, Sex, Age (months), Weight®

Parameter Estimates

Parameter	B	Std. Error	95% Wald Confidence Interval		Hypothesis Test
			Lower	Upper	Wald Chi-Square
(Intercept)	1.458	.0772	1.306	1.609	356.981
[bodysystemrecoded=2.00]	.109	.0948	-.077	.295	1.326
[bodysystemrecoded=3.00]	.040	.0549	-.068	.147	.526
[bodysystemrecoded=4.00]	.132	.1019	-.068	.332	1.678
[bodysystemrecoded=5.00]	.291	.0638	.166	.416	20.752
[bodysystemrecoded=6.00]	.328	.1084	.115	.540	9.138
[bodysystemrecoded=7.00]	-1.020	.1627	-1.339	-.701	39.306
[bodysystemrecoded=8.00]	-.396	.2332	-.853	.061	2.889
[bodysystemrecoded=10.00]	.190	.1885	-.179	.560	1.020
[bodysystemrecoded=11.00]	0[a]	.	.	.	.
[Indicated dose=1]	0[a]	.	.	.	.
[Indicated dose=2]	-.461	.0722	-.602	-.319	40.754
[Indicated dose=3]	0[a]	.	.	.	.
[Sex=0]	.137	.0465	.046	.228	8.652
[Sex=1]	.013	.0234	-.033	.059	.294
[Sex=2]	.047	.0366	-.025	.118	1.627
[Sex=3]	0[a]	.	.	.	.
Age (months)	-.001	.0002	-.001	.000	7.824
Weight®	-.005	.0009	-.007	-.003	31.056
(Scale)	.212[b]	.0069	.199	.226	

Parameter Estimates

Parameter	Hypothesis Test	
	df	Sig.
(Intercept)	1	.000
[bodysystemrecoded=2.00]	1	.249
[bodysystemrecoded=3.00]	1	.468
[bodysystemrecoded=4.00]	1	.195
[bodysystemrecoded=5.00]	1	<.001
[bodysystemrecoded=6.00]	1	.003
[bodysystemrecoded=7.00]	1	<.001
[bodysystemrecoded=8.00]	1	.089
[bodysystemrecoded=10.00]	1	.313
[bodysystemrecoded=11.00]	.	.
[Indicated dose=1]	.	.
[Indicated dose=2]	1	<.001
[Indicated dose=3]	.	.
[Sex=0]	1	.003
[Sex=1]	1	.588
[Sex=2]	1	.202
[Sex=3]	.	.
Age (months)	1	.005
Weight®	1	<.001
(Scale)		

Dependent Variable: mg/kg
Model: (Intercept), bodysystemrecoded, Indicated dose, Sex, Age (months), Weight®

a. Set to zero because this parameter is redundant.

b. Maximum likelihood estimate.

General Estimable Function

Parameter	Contrast					
	L1	L2	L3	L4	L5	L6
(Intercept)	1	0	0	0	0	0
[bodysystemrecoded=2.00]	0	1	0	0	0	0
[bodysystemrecoded=3.00]	0	0	1	0	0	0
[bodysystemrecoded=4.00]	0	0	0	1	0	0
[bodysystemrecoded=5.00]	0	0	0	0	1	0
[bodysystemrecoded=6.00]	0	0	0	0	0	1
[bodysystemrecoded=7.00]	0	0	0	0	0	0
[bodysystemrecoded=8.00]	0	0	0	0	0	0
[bodysystemrecoded=10.00]	0	0	0	0	0	0
[bodysystemrecoded=11.00]	1	- 1	- 1	- 1	- 1	- 1
[Indicated dose=1]	0	0	0	0	0	0
[Indicated dose=2]	0	0	0	0	0	0
[Indicated dose=3]	1	0	0	0	0	0
[Sex=0]	0	0	0	0	0	0
[Sex=1]	0	0	0	0	0	0
[Sex=2]	0	0	0	0	0	0
[Sex=3]	1	0	0	0	0	0
Age (months)	0	0	0	0	0	0
Weight®	0	0	0	0	0	0

General Estimable Function

Parameter	Contrast					
	L7	L8	L9	L12	L14	L15
(Intercept)	0	0	0	0	0	0
[bodysystemrecoded=2.00]	0	0	0	0	0	0
[bodysystemrecoded=3.00]	0	0	0	0	0	0
[bodysystemrecoded=4.00]	0	0	0	0	0	0
[bodysystemrecoded=5.00]	0	0	0	0	0	0
[bodysystemrecoded=6.00]	0	0	0	0	0	0
[bodysystemrecoded=7.00]	1	0	0	0	0	0
[bodysystemrecoded=8.00]	0	1	0	0	0	0
[bodysystemrecoded=10.00]	0	0	1	0	0	0
[bodysystemrecoded=11.00]	- 1	- 1	- 1	0	0	0
[Indicated dose=1]	1	0	0	0	0	0
[Indicated dose=2]	0	0	0	1	0	0
[Indicated dose=3]	- 1	0	0	- 1	0	0
[Sex=0]	0	0	0	0	1	0
[Sex=1]	0	0	0	0	0	1
[Sex=2]	0	0	0	0	0	0
[Sex=3]	0	0	0	0	- 1	- 1
Age (months)	0	0	0	0	0	0
Weight®	0	0	0	0	0	0

General Estimable Function

Parameter	Contrast		
	L16	L18	L19
(Intercept)	0	0	0
[bodysystemrecoded=2.00]	0	0	0
[bodysystemrecoded=3.00]	0	0	0
[bodysystemrecoded=4.00]	0	0	0
[bodysystemrecoded=5.00]	0	0	0
[bodysystemrecoded=6.00]	0	0	0
[bodysystemrecoded=7.00]	0	0	0
[bodysystemrecoded=8.00]	0	0	0
[bodysystemrecoded=10.00]	0	0	0
[bodysystemrecoded=11.00]	0	0	0
[Indicated dose=1]	0	0	0
[Indicated dose=2]	0	0	0
[Indicated dose=3]	0	0	0
[Sex=0]	0	0	0
[Sex=1]	0	0	0
[Sex=2]	1	0	0
[Sex=3]	- 1	0	0
Age (months)	0	1	0
Weight®	0	0	1

Dependent Variable: mg/kg
Model: (Intercept), bodysystemrecoded, Indicated dose, Sex, Age (months), Weight®
