# Supplementary material for: Prednisolone prescribing practices for dogs in Australia
Source: PLoS One. 2023 Feb 28;18(2):e0282440. doi: 10.1371/journal.pone.0282440 (PMC9974108; doi:10.1371/journal.pone.0282440)

Generalized Linear Models

Notes

Output Created		15-JUL-2022 17:20:55
Comments		
Input	Data	/Users/bonniepurcell/Desktop/SPSS /SPSS Whole data set correct sex.sav
	Active Dataset	DataSet1
	Filter	<none>
	Weight	<none>
	Split File	<none>
	N of Rows in Working Data File	2000
Missing Value Handling	Definition of Missing	User-defined missing values for factor, subject and within-subject variables are treated as missing.
	Cases Used	Statistics are based on cases with valid data for all variables in the model.
Weight Handling		not applicable
Syntax		GENLIN mgm2 BY bodysystemrecoded Indicateddose (ORDER=ASCENDING) WITH Weight® Agemonths /MODEL bodysystemrecoded Indicateddose Weight® Agemonths INTERCEPT=YES DISTRIBUTION=NORMAL LINK=IDENTITY /CRITERIA SCALE=MLE COVB=MODEL PCONVERGE=1E-006 (ABSOLUTE) SINGULAR=1E-012 ANALYSISTYPE=3(LR) CILEVEL=95 CITYPE=WALD LIKELIHOOD=FULL /MISSING CLASSMISSING=EXCLUDE /PRINT CPS DESCRIPTIVES MODELINFO FIT SUMMARY SOLUTION /SAVE MEANPRED COOK RESID.
Resources	Processor Time	00:00:00.48
	Elapsed Time	00:00:00.00

Notes

Variables Created or Modified	Predicted Value of the Mean of the Response	MeanPredicted
	Raw Residual	Residual
	Cook's Distance	CooksDistance

[DataSet1] /Users/bonniepurcell/Desktop/SPSS /SPSS Whole data set correct sex.sav

Model Information

Dependent Variable	mg/m2
Probability Distribution	Normal
Link Function	Identity

Case Processing Summary

	N	Percent
Included	1886	94.3%
Excluded	114	5.7%
Total	2000	100.0%

Categorical Variable Information

			N	Percent
Factor	bodysystemrecoded	2.00	24	1.3%
		3.00	80	4.2%
		4.00	22	1.2%
		5.00	61	3.2%
		6.00	33	1.7%
		7.00	10	0.5%
		8.00	7	0.4%
		10.00	6	0.3%
		11.00	1643	87.1%
		Total	1886	100.0%
	Indicated dose	1	10	0.5%
		2	1796	95.2%
		3	80	4.2%
		Total	1886	100.0%

Continuous Variable Information

		N	Minimum	Maximum	Mean
Dependent Variable	mg/m2	1886	1.09113813	106.655183	22.7675797
Covariate	Weight®	1886	1.56	90.00	19.3139
	Age (months)	1886	2	220	75.41

Continuous Variable Information

		Std. Deviation
Dependent Variable	mg/m2	13.0648639
Covariate	Weight®	12.57011
	Age (months)	49.441

Goodness of Fit[a]

	Value	df	Value/df
Deviance	270631.591	1874	144.414
Scaled Deviance	1886.000	1874	
Pearson Chi-Square	270631.591	1874	144.414
Scaled Pearson Chi-Square	1886.000	1874	
Log Likelihood[b]	-7359.339		
Akaike's Information Criterion (AIC)	14744.678		
Finite Sample Corrected AIC (AICC)	14744.873		
Bayesian Information Criterion (BIC)	14816.727		
Consistent AIC (CAIC)	14829.727		

Dependent Variable: mg/m2
Model: (Intercept), bodysystemrecoded, Indicated dose, Weight®, Age (months)[a]

a. Information criteria are in smaller-is-better form.

b. The full log likelihood function is displayed and used in computing information criteria.

Omnibus Test[a]

Likelihood Ratio Chi-Square	df	Sig.
326.320	11	.000

Dependent Variable: mg/m2
Model: (Intercept), bodysystemrecoded, Indicated dose, Weight®, Age (months)[a]

a. Compares the fitted model against the intercept-only...

Tests of Model Effects

	Type III		
Source	Likelihood Ratio Chi-Square	df	Sig.
(Intercept)	393.523	1	.000
bodysystemrecoded	24.993	7	<.001
Indicated dose	37.137	1	<.001
Weight®	138.359	1	.000
Age (months)	11.390	1	<.001

Dependent Variable: mg/m2
Model: (Intercept), bodysystemrecoded, Indicated dose, Weight®, Age (months)

Parameter Estimates

Parameter	B	Std. Error	95% Wald Confidence Interval		Hypothesis Test
			Lower	Upper	Wald Chi-Square
(Intercept)	29.489	1.9592	25.649	33.329	226.556
[bodysystemrecoded=2.00]	2.466	2.4648	-2.365	7.297	1.001
[bodysystemrecoded=3.00]	1.657	1.4181	-1.122	4.437	1.366
[bodysystemrecoded=4.00]	4.776	2.5896	-.299	9.852	3.402
[bodysystemrecoded=5.00]	4.556	1.6540	1.315	7.798	7.589
[bodysystemrecoded=6.00]	11.231	2.7617	5.818	16.644	16.538
[bodysystemrecoded=7.00]	-24.155	4.2257	-32.438	-15.873	32.676
[bodysystemrecoded=8.00]	4.453	4.6550	-4.671	13.576	.915
[bodysystemrecoded=10.00]	3.178	4.9032	-6.432	12.788	.420
[bodysystemrecoded=11.00]	0[a]	.	.	.	.
[Indicated dose=1]	0[a]	.	.	.	.
[Indicated dose=2]	-11.390	1.8598	-15.035	-7.744	37.505
[Indicated dose=3]	0[a]	.	.	.	.
Weight®	.271	.0226	.227	.316	143.561
Age (months)	-.020	.0059	-.032	-.008	11.424
(Scale)	143.495[b]	4.6728	134.623	152.952	

Parameter Estimates

Parameter	Hypothesis Test	
	df	Sig.
(Intercept)	1	.000
[bodysystemrecoded=2.00]	1	.317
[bodysystemrecoded=3.00]	1	.243
[bodysystemrecoded=4.00]	1	.065
[bodysystemrecoded=5.00]	1	.006
[bodysystemrecoded=6.00]	1	<.001
[bodysystemrecoded=7.00]	1	<.001
[bodysystemrecoded=8.00]	1	.339
[bodysystemrecoded=10.00]	1	.517
[bodysystemrecoded=11.00]	.	.
[Indicated dose=1]	.	.
[Indicated dose=2]	1	<.001
[Indicated dose=3]	.	.
Weight®	1	.000
Age (months)	1	<.001
(Scale)		

Dependent Variable: mg/m2
Model: (Intercept), bodysystemrecoded, Indicated dose, Weight®, Age (months)

a. Set to zero because this parameter is redundant.

b. Maximum likelihood estimate.

GGraph

Notes

Output Created		15-JUL-2022 17:21:35
Comments		
Input	Data	/Users/bonniepurcell/Desktop/SPSS /SPSS Whole data set correct sex.sav
	Active Dataset	DataSet1
	Filter	<none>
	Weight	<none>
	Split File	<none>
	N of Rows in Working Data File	2000
Syntax		GGRAPH<br>/GRAPHDATASET NAME="graphdataset" VARIABLES=Residual MISSING=LISTWISE REPORTMISSING=NO<br>/GRAPHSPEC SOURCE=INLINE.<br>BEGIN GPL<br>SOURCE: s=userSource(id("graphdataset"))<br>DATA: Residual=col(source(s), name("Residual"))<br>GUIDE: axis(dim(1), label("Raw Residual"))<br>GUIDE: axis(dim(2), label("Frequency"))<br>GUIDE: text.title(label("Simple Histogram of Raw Residual"))<br>ELEMENT: interval(position(summary.count(bin.rect(Residual))), shape.interior(shape.square))<br>END GPL.
Resources	Processor Time	00:00:02.56
	Elapsed Time	00:00:03.00

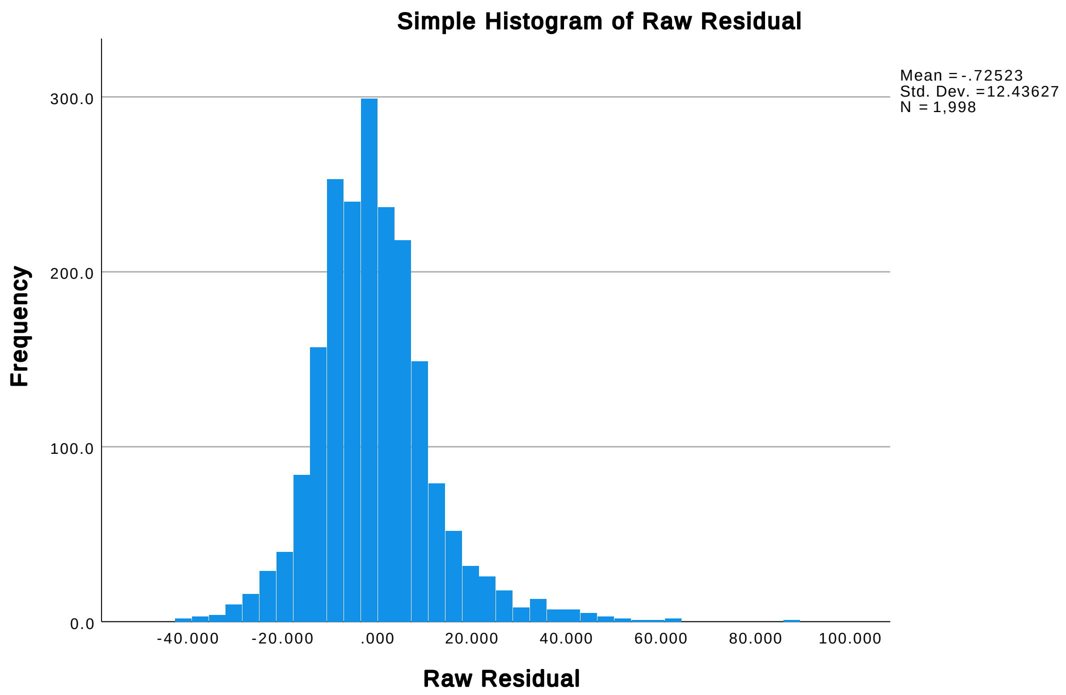

GGraph

Notes

Output Created		15-JUL-2022 17:22:45
Comments		
Input	Data	/Users/bonniepurcell/Desktop/SPSS /SPSS Whole data set correct sex.sav
	Active Dataset	DataSet1
	Filter	<none>
	Weight	<none>
	Split File	<none>
	N of Rows in Working Data File	2000

Notes

Syntax		GGRAPH<br>/GRAPHDATASET NAME="graphdataset" VARIABLES=CooksDistance mgm2 MISSING=LISTWISE REPORTMISSING=NO<br>/GRAPHSPEC SOURCE=INLINE<br>/FITLINE TOTAL=NO SUBGROUP=NO.<br>BEGIN GPL<br>SOURCE: s=userSource(id("graphdataset"))<br>DATA: CooksDistance=col(source(s), name("CooksDistance"))<br>DATA: mgm2=col(source(s), name("mgm2"))<br>GUIDE: axis(dim(1), label("Cook's Distance"))<br>GUIDE: axis(dim(2), label("mg/m2"))<br>GUIDE: text.title(label("Scatter Plot of mg/m2 by Cook's Distance"))<br>ELEMENT: point(position(CooksDistance*mgm2))<br>END GPL.
Resources	Processor Time	00:00:00.19
	Elapsed Time	00:00:00.00

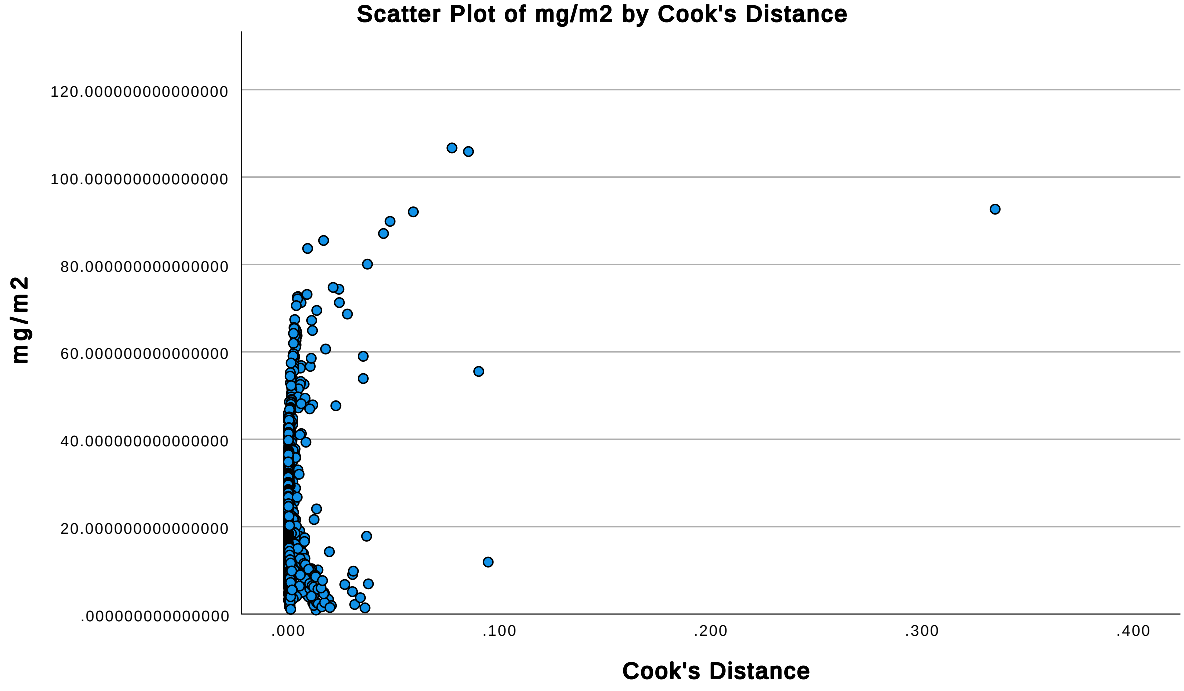

GGraph

Notes

Output Created		15-JUL-2022 17:23:28
Comments		
Input	Data	/Users/bonniepurcell/Desktop/SPSS /SPSS Whole data set correct sex.sav
	Active Dataset	DataSet1
	Filter	<none>
	Weight	<none>
	Split File	<none>
	N of Rows in Working Data File	2000
Syntax		GGRAPH<br>/GRAPHDATASET NAME="graphdataset" VARIABLES=mgm2 CooksDistance MISSING=LISTWISE REPORTMISSING=NO<br>/GRAPHSPEC SOURCE=INLINE<br>/FITLINE TOTAL=NO SUBGROUP=NO.<br>BEGIN GPL<br>SOURCE: s=userSource(id("graphdataset"))<br>DATA: mgm2=col(source(s), name("mgm2"))<br>DATA: CooksDistance=col(source(s), name("CooksDistance"))<br>GUIDE: axis(dim(1), label("mg/m2"))<br>GUIDE: axis(dim(2), label("Cook's Distance"))<br>GUIDE: text.title(label("Scatter Plot of Cook's Distance by mg/m2"))<br>ELEMENT: point(position(mgm2*CooksDistance))<br>END GPL.
Resources	Processor Time	00:00:00.15
	Elapsed Time	00:00:00.00

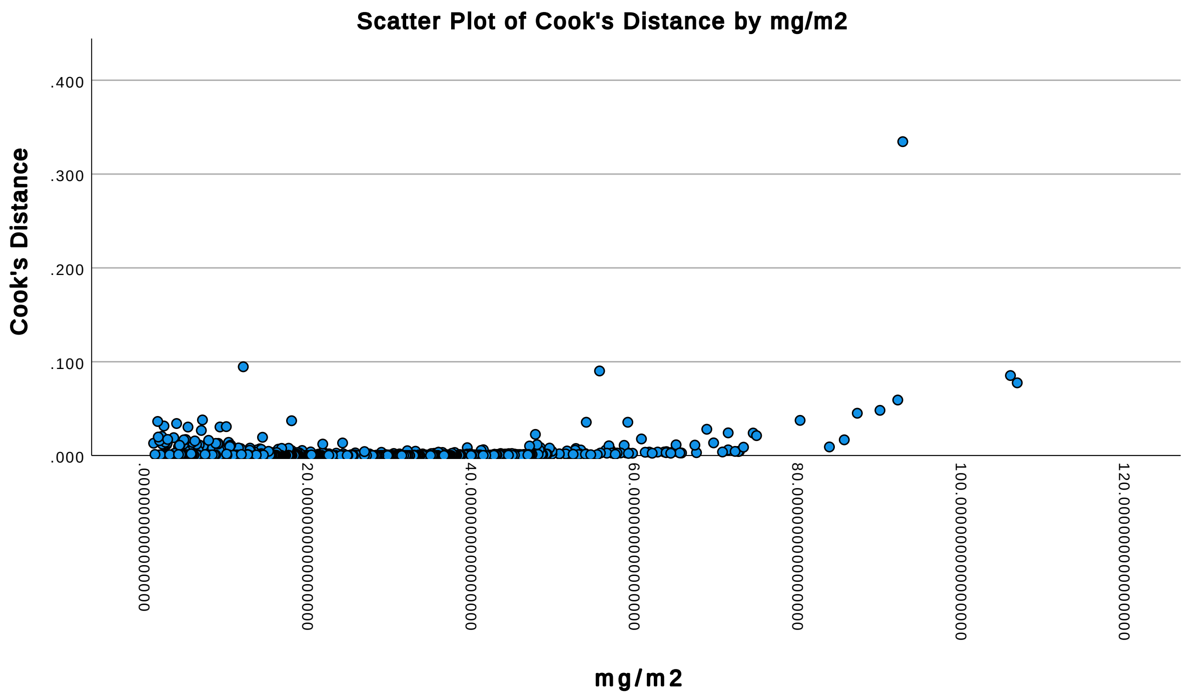

GGraph

Notes

Output Created		15-JUL-2022 17:24:12
Comments		
Input	Data	/Users/bonniepurcell/Desktop/SPSS /SPSS Whole data set correct sex.sav
	Active Dataset	DataSet1
	Filter	<none>
	Weight	<none>
	Split File	<none>
	N of Rows in Working Data File	2000

Notes

Syntax		GGRAPH<br>/GRAPHDATASET NAME="graphdataset" VARIABLES=mgm2 MeanPredicted MISSING=LISTWISE REPORTMISSING=NO<br>/GRAPHSPEC SOURCE=INLINE<br>/FITLINE TOTAL=NO.<br>BEGIN GPL<br>SOURCE: s=userSource (id("graphdataset"))<br>DATA: mgm2=col (source(s), name ("mgm2"))<br>DATA: MeanPredicted=col (source(s), name ("MeanPredicted"))<br>GUIDE: axis(dim(1), label("mg/m2"))<br>GUIDE: axis(dim(2), label("Predicted Value of Mean of Response"))<br>GUIDE: text.title(label ("Scatter Plot of Predicted Value of Mean of Response by mg/m2"))<br>ELEMENT: point(position (mgm2*MeanPredicted))<br>END GPL.
Resources	Processor Time	00:00:00.15
	Elapsed Time	00:00:00.00

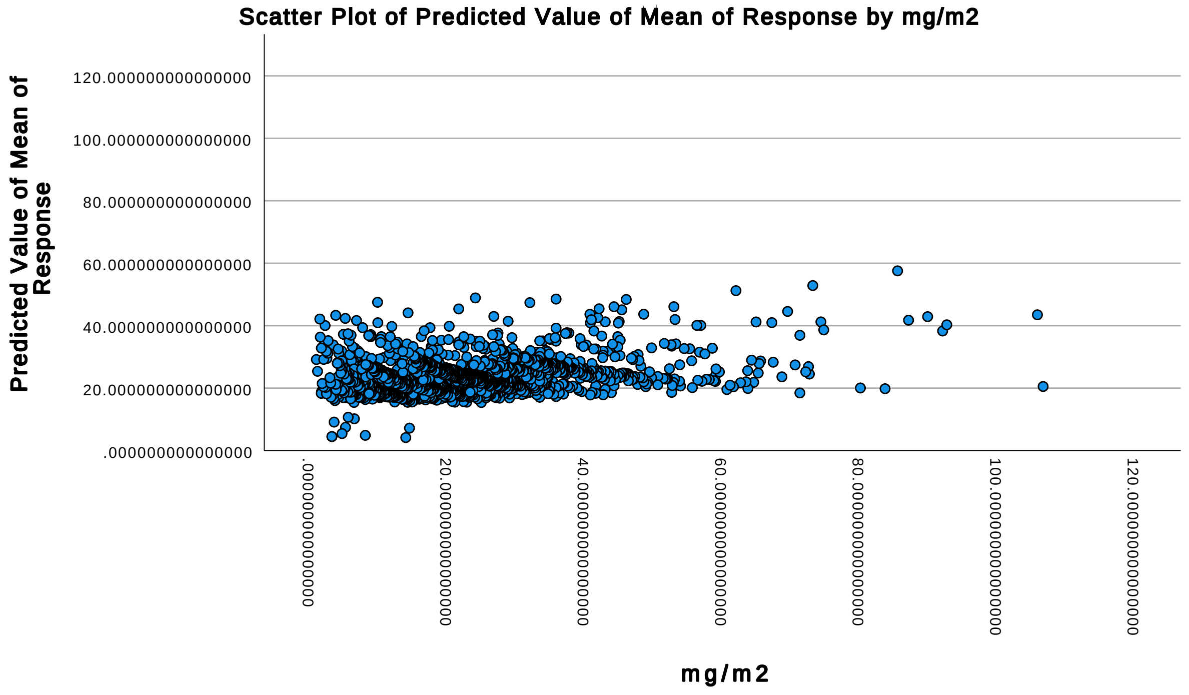

Generalized Linear Models

Notes

Output Created		15-JUL-2022 17:29:11
Comments		
Input	Data	/Users/bonniepurcell/Desktop/SPSS /SPSS Whole data set correct sex.sav
	Active Dataset	DataSet1
	Filter	CooksDistance <= 0.05 (FILTER)
	Weight	<none>
	Split File	<none>
	N of Rows in Working Data File	1992
Missing Value Handling	Definition of Missing	User-defined missing values for factor, subject and within-subject variables are treated as missing.
	Cases Used	Statistics are based on cases with valid data for all variables in the model.
Weight Handling		not applicable
Syntax		GENLIN mgm2 BY bodysystemrecoded Indicateddose (ORDER=ASCENDING) WITH Weight® Agemonths /MODEL bodysystemrecoded Indicateddose Weight® Agemonths INTERCEPT=YES DISTRIBUTION=NORMAL LINK=IDENTITY /CRITERIA SCALE=MLE COVB=MODEL PCONVERGE=1E-006 (ABSOLUTE) SINGULAR=1E-012 ANALYSISTYPE=3(LR) CILEVEL=95 CITYPE=WALD LIKELIHOOD=FULL /MISSING CLASSMISSING=EXCLUDE /PRINT CPS DESCRIPTIVES MODELINFO FIT SUMMARY SOLUTION /SAVE MEANPRED COOK RESID.
Resources	Processor Time	00:00:00.43
	Elapsed Time	00:00:00.00

Notes

Variables Created or Modified	Predicted Value of the Mean of the Response	MeanPredicted_1
	Raw Residual	Residual_1
	Cook's Distance	CooksDistance_1

Model Information

Dependent Variable	mg/m2
Probability Distribution	Normal
Link Function	Identity

Case Processing Summary

	N	Percent
Included	1880	94.4%
Excluded	112	5.6%
Total	1992	100.0%

Categorical Variable Information

			N	Percent
Factor	bodysystemrecoded	2.00	24	1.3%
		3.00	79	4.2%
		4.00	22	1.2%
		5.00	60	3.2%
		6.00	32	1.7%
		7.00	10	0.5%
		8.00	4	0.2%
		10.00	6	0.3%
		11.00	1643	87.4%
		Total	1880	100.0%
	Indicated dose	1	10	0.5%
		2	1794	95.4%
		3	76	4.0%
		Total	1880	100.0%

Continuous Variable Information

		N	Minimum	Maximum	Mean
Dependent Variable	mg/m2	1880	1.09113813	89.8525891	22.5931278
Covariate	Weight®	1880	1.56	90.00	19.3097
	Age (months)	1880	2	220	75.46

Continuous Variable Information

		Std. Deviation
Dependent Variable	mg/m2	12.5699326
Covariate	Weight®	12.58103
	Age (months)	49.421

Goodness of Fit[a]

	Value	df	Value/df
Deviance	251209.031	1868	134.480
Scaled Deviance	1880.000	1868	
Pearson Chi-Square	251209.031	1868	134.480
Scaled Pearson Chi-Square	1880.000	1868	
Log Likelihood[b]	-7268.917		
Akaike's Information Criterion (AIC)	14563.834		
Finite Sample Corrected AIC (AICC)	14564.030		
Bayesian Information Criterion (BIC)	14635.842		
Consistent AIC (CAIC)	14648.842		

Dependent Variable: mg/m2
Model: (Intercept), bodysystemrecoded, Indicated dose, Weight®, Age (months)[a]

a. Information criteria are in smaller-is-better form.

b. The full log likelihood function is displayed and used in computing information criteria.

Omnibus Test[a]

Likelihood Ratio Chi-Square	df	Sig.
314.091	11	.000

Dependent Variable: mg/m2
Model: (Intercept), bodysystemrecoded, Indicated dose, Weight®, Age (months)[a]

a. Compares the fitted model against the intercept-only...

Tests of Model Effects

Source	Type III		
	Likelihood Ratio Chi-Square	df	Sig.
(Intercept)	335.722	1	.000
bodysystemrecoded	24.205	7	.001
Indicated dose	32.280	1	<.001
Weight®	149.497	1	.000
Age (months)	8.884	1	.003

Dependent Variable: mg/m2
Model: (Intercept), bodysystemrecoded, Indicated dose, Weight®, Age (months)

Parameter Estimates

Parameter	B	Std. Error	95% Wald Confidence Interval		Hypothesis Test
			Lower	Upper	Wald Chi-Square
(Intercept)	28.277	1.9223	24.510	32.045	216.400
[bodysystemrecoded=2.00]	2.405	2.3786	-2.257	7.067	1.023
[bodysystemrecoded=3.00]	.399	1.3768	-2.299	3.098	.084
[bodysystemrecoded=4.00]	4.630	2.4990	-.268	9.528	3.433
[bodysystemrecoded=5.00]	3.801	1.6038	.658	6.944	5.617
[bodysystemrecoded=6.00]	10.132	2.7036	4.833	15.430	14.044
[bodysystemrecoded=7.00]	-23.279	4.0884	-31.292	-15.266	32.421
[bodysystemrecoded=8.00]	-7.949	5.8549	-19.425	3.526	1.843
[bodysystemrecoded=10.00]	3.255	4.7315	-6.019	12.528	.473
[bodysystemrecoded=11.00]	0[a]	.	.	.	.
[Indicated dose=1]	0[a]	.	.	.	.
[Indicated dose=2]	-10.396	1.8219	-13.967	-6.825	32.559
[Indicated dose=3]	0[a]	.	.	.	.
Weight®	.273	.0219	.230	.315	155.602
Age (months)	-.017	.0057	-.028	-.006	8.905
(Scale)	133.622[b]	4.3583	125.347	142.443	

Parameter Estimates

Parameter	Hypothesis Test	
	df	Sig.
(Intercept)	1	.000
[bodysystemrecoded=2.00]	1	.312
[bodysystemrecoded=3.00]	1	.772
[bodysystemrecoded=4.00]	1	.064
[bodysystemrecoded=5.00]	1	.018
[bodysystemrecoded=6.00]	1	<.001
[bodysystemrecoded=7.00]	1	<.001
[bodysystemrecoded=8.00]	1	.175
[bodysystemrecoded=10.00]	1	.492
[bodysystemrecoded=11.00]	.	.
[Indicated dose=1]	.	.
[Indicated dose=2]	1	<.001
[Indicated dose=3]	.	.
Weight®	1	.000
Age (months)	1	.003
(Scale)		

Dependent Variable: mg/m2
Model: (Intercept), bodysystemrecoded, Indicated dose, Weight®, Age (months)

a. Set to zero because this parameter is redundant.

b. Maximum likelihood estimate.

GGraph

Notes

Output Created		15-JUL-2022 17:30:04
Comments		
Input	Data	/Users/bonniepurcell/Desktop/SPSS /SPSS Whole data set correct sex.sav
	Active Dataset	DataSet1
	Filter	CooksDistance <= 0.05 (FILTER)
	Weight	<none>
	Split File	<none>
	N of Rows in Working Data File	1992
Syntax		GGRAPH<br>/GRAPHDATASET NAME="graphdataset" VARIABLES=Residual_1 MISSING=LISTWISE REPORTMISSING=NO<br>/GRAPHSPEC SOURCE=INLINE.<br>BEGIN GPL<br>SOURCE: s=userSource(id("graphdataset"))<br>DATA: Residual_1=col(source(s), name("Residual_1"))<br>GUIDE: axis(dim(1), label("Raw Residual"))<br>GUIDE: axis(dim(2), label("Frequency"))<br>GUIDE: text.title(label("Simple Histogram of Raw Residual"))<br>ELEMENT: interval(position(summary.count(bin.rect(Residual_1))), shape.interior(shape.square))<br>END GPL.
Resources	Processor Time	00:00:00.16
	Elapsed Time	00:00:00.00

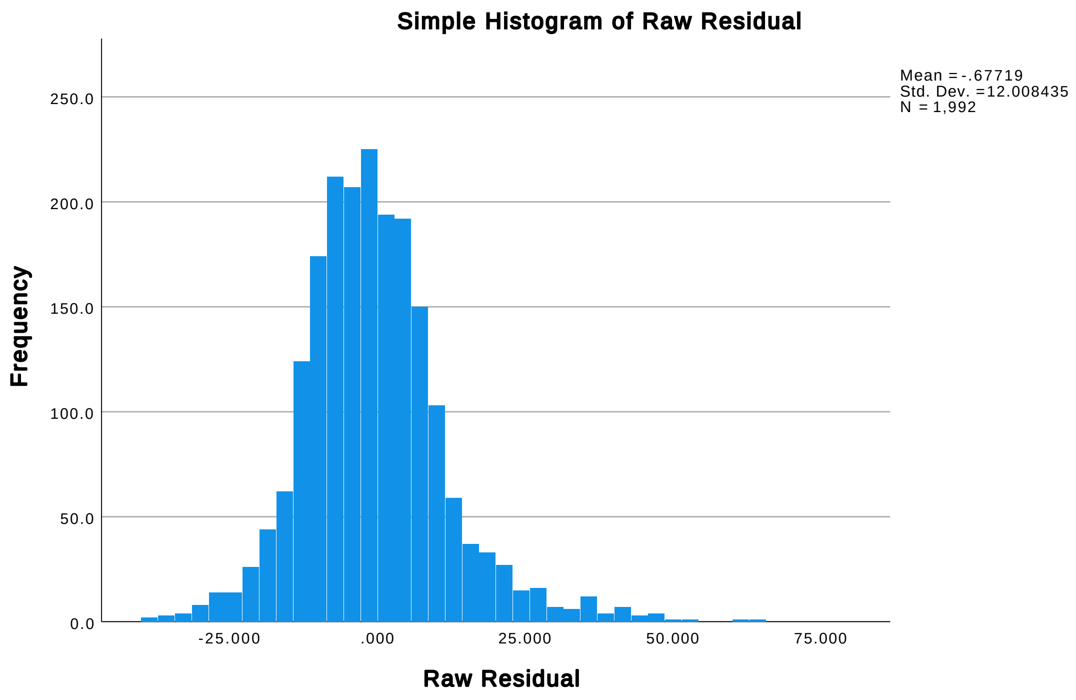

GGraph

Notes

Output Created		15-JUL-2022 17:31:30
Comments		
Input	Data	/Users/bonniepurcell/Desktop/SPSS /SPSS Whole data set correct sex.sav
	Active Dataset	DataSet1
	Filter	CooksDistance <= 0.05 (FILTER)
	Weight	<none>
	Split File	<none>
	N of Rows in Working Data File	1992

Notes

Syntax		GGRAPH<br>/GRAPHDATASET NAME="graphdataset" VARIABLES=mgm2 MeanPredicted_1 MISSING=LISTWISE REPORTMISSING=NO<br>/GRAPHSPEC SOURCE=INLINE<br>/FITLINE TOTAL=NO SUBGROUP=NO.<br>BEGIN GPL<br>SOURCE: s=userSource (id("graphdataset"))<br>DATA: mgm2=col (source(s), name ("mgm2"))<br>DATA: MeanPredicted_1=col (source(s), name ("MeanPredicted_1"))<br>GUIDE: axis(dim(1), label("mg/m2"))<br>GUIDE: axis(dim(2), label("Predicted Value of Mean of Response"))<br>GUIDE: text.title(label ("Scatter Plot of Predicted Value of Mean of Response by mg/m2"))<br>ELEMENT: point(position (mgm2*MeanPredicted_1))<br>END GPL.
Resources	Processor Time	00:00:00.13
	Elapsed Time	00:00:00.00

Scatter Plot of Predicted Value of Mean of Response by mg/m2

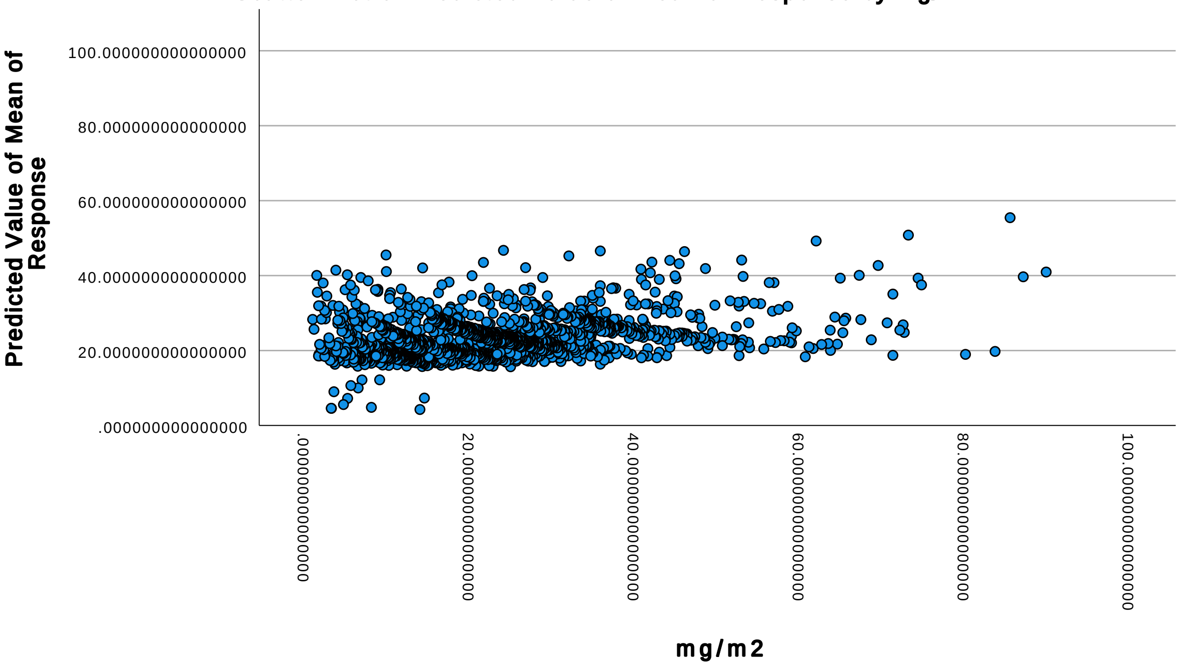

Supplement: S4 File — (PDF) [file pone.0282440.s007.pdf]
