# Supplementary material for: Prednisolone prescribing practices for dogs in Australia
Source: PLoS One. 2023 Feb 28;18(2):e0282440. doi: 10.1371/journal.pone.0282440 (PMC9974108; doi:10.1371/journal.pone.0282440)
Supplement: Attachment — Submitted filename: Response to reviewers.pdf [file pone.0282440.s008.pdf]

To Mark Zabel and reviewers of “Prednisolone prescribing practices for dogs in Australia”,

Thank you for considering our manuscript and the constructive feedback provided. Please find below our responses to the points raised during the review process.

Reviewer #1, comment 1: We have changed “Evan’s syndrome” to “Concurrent immune mediated haemolytic anaemia and immune mediated thrombocytopenia” in Table 5. This is clearer and more correct.

Reviewer #1, comment 2: There is no clear explanation for female entire dogs receiving higher doses than male neutered dogs in the mg/kg multivariable analysis. Noting that it is a relatively small increase, with a wide confidence interval, and that female entire dogs represent the smallest group (n = 114), we wanted to avoid over interpreting this result and so did not discuss it extensively. We have now added that this may be a type 1 error, given it was not found in the mg/m^2 multivariable analysis.

Reviewer #2, comment 1: We’re not quite sure, but think that this comment relates to the categories being not mutually exclusive in Table 3. Thank you for picking this up. We have revised the table and double-checked the text to ensure that the categories are now consistently defined as physiological (< 0.3 mg/kg), anti-inflammatory (0.3 – 1.0 mg/kg), intermediate (> 1.0 - < 1.5 mg/kg) and immunosuppressive (≥ 1.5 mg/kg) throughout the manuscript.

Reviewer #2, comment 2: Line 248 has been amended as suggested

As an additional change, to improve clarity, we have changed the wording of one line in the discussion for clarity. This is line 385-386, regarding large dogs prescribed prednisolone at 50 mg/m^2/day. The previous wording could have been misinterpreted and we think the new wording is clearer, pointing out that 67% of large dogs, receiving that dose, did so for inflammatory disease conditions. This can be reverted to the original wording if this is preferred.

Finally, we have changed the address for the Melbourne Veterinary School to the Faculty of Science, due to a recent departmental change.

Thank you again for your time and consideration,

Bonnie Purcell, Julien Dandrieux and Anke Wiethoelter.
